# Understanding the Aggregation of Model Island and Archipelago Asphaltene Molecules near Kaolinite Surfaces using Molecular Dynamics

**DOI:** 10.1021/acs.energyfuels.3c00504

**Published:** 2023-07-28

**Authors:** Azeezat Ali, David R. Cole, Alberto Striolo

**Affiliations:** † Department of Chemical Engineering, University College London, London WC1E 6BT, United Kingdom; ‡ School of Earth Sciences, The Ohio State University, Columbus, Ohio 43210, United States; § School of Sustainable Chemical, Biological and Materials Engineering, The University of Oklahoma, Norman, Oklahoma 73019, United States

## Abstract

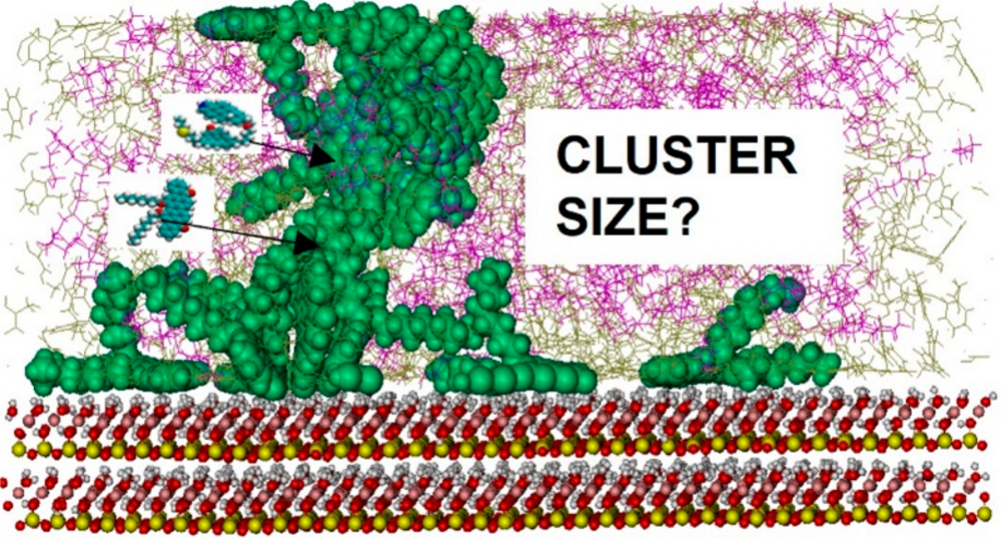

The solubility of asphaltenes in hydrocarbons changes with pressure, composition, and temperature, leading to precipitation and deposition, thereby causing one of the crucial problems that negatively affects oil production, transportation, and processing. Because, in some circumstances, it might be advantageous to promote asphaltene agglomeration into small colloidal particles, molecular dynamics simulations were conducted here to understand the impacts of a chemical additive inspired by cyclohexane on the mechanism of aggregation of model island and archipelago asphaltene molecules in toluene. We compared the results in the presence and absence of a kaolinite surface at 300 and 400 K. Cluster size analyses, radial distribution functions, angles between asphaltenes, radius of gyration, and entropic and energetic calculations were used to provide insights on the behavior of these systems. The results show that the hypothetical additive inspired by cyclohexane promoted the aggregation of both asphaltenes. Structural differences were observed among the aggregates obtained in our simulations. These differences are attributed to the number of aromatic cores and side chains on the asphaltene molecules as well as to that of heteroatoms. For the island structure, aggregation in the bulk phase was less pronounced than that in the proximity of the kaolinite surface, whereas the opposite was observed for the archipelago structure. In both cases, the additive promoted stacking of asphaltenes, yielding more compact aggregates. The results provided insights into the complex nature of asphaltene aggregation, although computational approaches that can access longer time and larger size scales should be chosen for quantifying emergent meso- and macroscale properties of systems containing asphaltenes in larger numbers than those that can currently be sampled via atomistic simulations.

## 1 Introduction

Asphaltenes, the heaviest and most polar fraction of crude oil, pose a flow assurance problem in the oil industry due to their tendency to form aggregates upon changes in temperature, pressure, and the physical-chemical environment. The resultant aggregates can clog wells, pipelines, surface facilities, and subsurface formations, leading to a significant reduction in productivity, as well as to possible safety issues.^1−5^ Asphaltenes are generally defined as a solubility class depending on their solubility in aromatic solvents (such as toluene and xylene) and insolubility in aliphatic solvents (such as *n*-pentane and *n*-heptane).^6,7^ As a consequence, their molecular structure is highly variable, and it strongly depends on the crude oil of interest.

In general, asphaltenes are represented either by the island structure, consisting of polycyclic aromatic hydrocarbon (PAH) rings attached to aliphatic chains, or by the archipelago structure, which consists of several aromatic cores loosely bonded by aliphatic chains. Chemical analysis has shown that PAH cores can contain heteroatoms such as nitrogen, oxygen, and sulfur, which often control aggregation,^8−11^ and sometimes lead to hydrogen bonds.^9,12^

To interrogate the mechanism of asphaltene precipitation, molecular simulation studies have been attempted. Of these, several molecular dynamics (MD) studies showed that asphaltenes aggregate assuming face-to-face (parallel), T-shaped, or offset stacking structures. Interactions between PAH cores (π–π stacking) are frequently identified as the main driving force for aggregation. The size of the PAH cores controls the formation of π stacks and other aggregate types. Higher aromaticity and shorter side chains lead to greater aggregation.^13−17^

In addition to aggregation in the bulk, many practical applications are also affected by asphaltene adsorption on surfaces, a process likely controlled by both surface features and asphaltene characteristics. Adsorption of asphaltenes has been documented on mineral surfaces such as calcite, mica, and silica,^18−26^ which affects both asphaltene aggregation and wettability of the mineral surfaces. Liu et al.^20,27^ concluded from their experiments and MD simulations that polar silica surfaces are more attractive to asphaltene than nonpolar silica. They showed that the arrangement of heteroatoms on asphaltene contributes to the strength and mechanism of their adsorption. Xiong et al.^28^ determined that the asphaltene model they used adsorbs on silica surface in heptane through hydrogen bonds with the hydroxyl groups on the surface, whereas in toluene, aggregates form and mostly stay in the bulk oil phase. Compared to silica, adsorption on muscovite is weaker and the molecules adsorb somewhat perpendicularly to the surface by interacting with the polar groups in the side chains or the edge of the PAHs.^29^ Mohammed and Gadikota^18,30^ revealed that asphaltenes adsorb as small nanoaggregates on calcite and silica surfaces compared to the aggregates observed in bulk systems. The authors also demonstrated that CO_2_ increases the aggregation of asphaltenes while displacing them from calcite due to the strong affinity between CO_2_ and the surface. Fang et al.^31^ observed, via MD simulations, that CO_2_ causes asphaltenes to first form dimers and then aggregate and adsorb onto silica surfaces. Increasing pressure was found to weaken the interactions between asphaltenes and their adsorption on silica. Li et al.^32^ experimentally observed CO_2_-induced asphaltene deposition on quartz. The aggregation and deposition were enhanced by increases in the temperature, resulting in a less water-wet surface. Li et al.^33^ also reported that flooding with CO_2_ (as opposed to flooding with methane and propane) leads to high asphaltene aggregation and found that the degree of aggregation increases with decreasing temperature. On quartz, the time taken for Violanthrone-79 (VO-79), a PAH frequently used as a model for asphaltene,^34^ to adsorb on the surface increased with temperature, even though the number of stably adsorbed VO-79 molecules remained relatively constant.^35^ Moreover, the adsorption was more stable in heptane than in toluene, which allowed the PAH to desorb back into the bulk.^35^ Nassar et al.^24^ found that asphaltene adsorbs more strongly on acidic alumina surfaces than on basic or neutral ones. These studies highlight the important contribution of the chemical composition of mineral surfaces in determining asphaltene precipitation.

To control and possibly prevent asphaltene precipitation, chemical inhibitors and dispersants are commonly used. Simulations have been utilized in identifying the mechanistic behavior of these additives. For example, employing MD simulations, Headen and Boek^36^ showed that the presence of 50 wt % limonene reduces the aggregation of asphaltene in CO_2_. Alkylphenols have also been identified as possible inhibitors.^37−40^ According to Aminzadeh et al.,^37^ nonylphenols slightly reduce the size of asphaltene aggregates, with stronger effects resulting from nonylphenol added at the nucleation stage as opposed to being added earlier. Further increases in the concentration of nonylphenol can lead to their aggregation, a result ascribed to high dipole moments and the ability to form hydrogen bonds. Octylphenol also reduces the interaction energies and hydrogen bonds between asphaltenes. However, in terms of aggregation number, this inhibitor only delays asphaltene aggregation, rather than preventing it.^38^ The experiments reported by Lin et al.^40^ showed that alkylphenols with longer alkyl tails increase the steric repulsion between aggregates, although the dispersants reduce the aggregate size leading to higher deposition rate. Strong repulsive forces between asphaltenes lead to the formation of “softer” aggregates that are readily eroded when shear flow is applied after deposition. Using MD, Jiang et al.^41^ found that dodecylbenzenesulfonic acid (DBSA) reduces the rate and extent of asphaltene aggregation by reducing π–π stacking. When added after aggregation, DBSA weakens the aggregates by breaking the polar group bonds between the molecules but not the π–π stacking. Another study explored the effects of temperature on the performance of chemical inhibitors dodecyl benzenesulfonic acid (DBSA), benzoic acid (BA), and salicylic acid (SA) in the temperature range between 25 and 65 °C. DBSA was found to be the most effective inhibitor due to its acid–base interactions with asphaltenes. The increase in temperature accelerated asphaltene aggregation, which reduced the effectiveness of inhibitors. Salicylic acid caused the aggregates to increase at 65 °C as a result of strong hydrogen bonds.^42^ The results obtained for salicylic acid are comparable to those reported by Madhi et al.,^43^ where increasing the concentrations of cetyltrimethylammonium bromide (CTAB) and sodium dodecyl sulfate (SDS) led to self-assembly of the inhibitors, reducing their effectiveness. These studies, in general, show that the efficacy of a chemical inhibitor is determined by the strength of the interactions between asphaltenes and inhibitors relative to those between the two inhibitors. How this balance will be affected by the presence of a surface has not yet been fully quantified.

Despite this extensive body of work, the mechanism of action of asphaltene inhibitors is not well understood. For example, Kuang et al.^44^ suggested that although conventional dispersants reduce the size of asphaltene aggregates, they do not necessarily alleviate asphaltene deposition. This leads to the possibility that enhancing the aggregation of asphaltenes into small structures could be a better strategy for preventing large plugs. Accordingly, the research presented here explores, on a molecular level, the use of a hydrocarbon chain to increase the aggregation of two asphaltene structures (island and archipelago), in combination with the proximity to a kaolinite surface. Kaolinite, a clay, is chosen because it is one of the common minerals found in reservoirs.^45,46^ Kaolinite is a layered aluminosilicate with the chemical formula Al_2_O_3_2SiO_2_·2H_2_O. It has a 1:1 uncharged layered structure, consisting of alternating sheets of silica (SiO_4_) tetrahedra and octahedral alumina oxyhydroxides, joined by apical oxygen atoms. In our simulations, kaolinite was cleaved along the 001 basal plane, normal to the Z axis, to create the alumina surface terminated with a plane of surface hydroxyl groups.^47−49^ We employ MD simulations at the atomic resolution for this investigation.

The outline of this article is as follows: In the next section, we show the hydrocarbon models used and describe the force fields, initial configurations, and simulation algorithms. In section 3, we analyze the results by quantifying properties such as aggregate sizes, radial distribution functions, angles between the asphaltene molecules, and entropy analysis. Finally, we summarize in the conclusions our main findings and suggest improvements to our method.

## 2 Simulation Methodology

### Molecular Models

**Figure 1:**
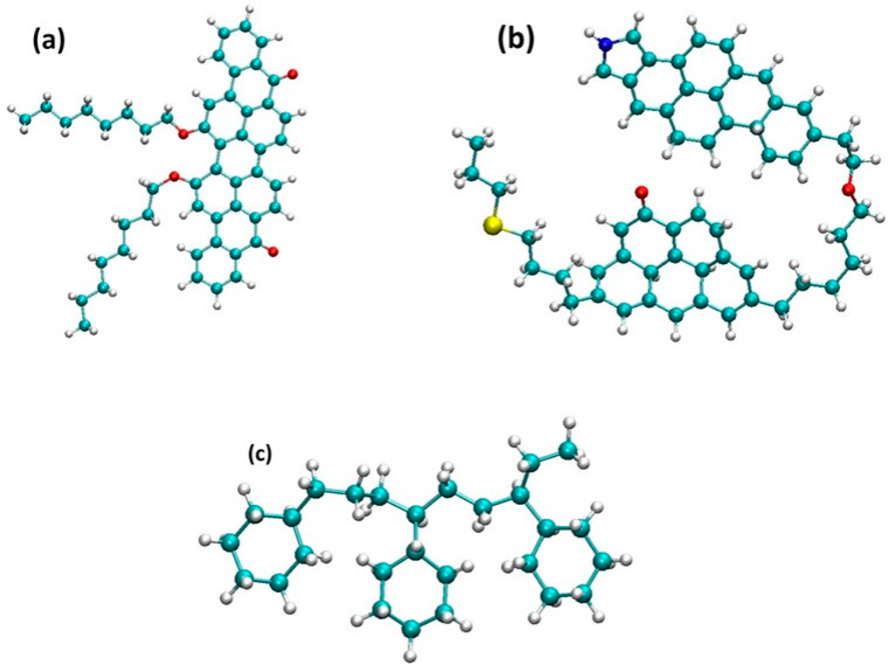
Models of asphaltene molecules used in this study: (a) island structure, (b) archipelago structure, and (c) cyclohexane chain. Cyan = carbon; white = hydrogen; red = oxygen; yellow = nitrogen; and blue = sulfur.

In Figure 1, we present the asphaltene models used in this study. Violanthrone-79 (712.9 g/mol),^29,50^ which consists of a large central poly aromatic core connected to two aliphatic side chains, represents the island structure (indicated as ASPH in what follows, shown in Figure 1a). This compound has been used experimentally to conduct controlled tests; it shows a tendency to aggregate in heptane to a larger extent than in toluene, which supports the choice of Violanthrone-79 as a model for asphaltene.^51^ As for the representative archipelago (ARCH) structure, a molecule with two aromatic cores, with molecular weight 792.2 g/mol, was chosen (Figure 1b).^52^ Although there have been several models proposed for asphaltenes, we chose to focus on one island model and one archipelago model to test the effects of the cyclohexane oligomer as a proof of principle for the potential of a putative chemical additive to increase the aggregation of asphaltenes with different structures. Of note, Violanthrone-79 is a real polycyclic aromatic compound, allowing our results to potentially be compared to experiments. Because previous research showed that asphaltenes are almost insoluble in cyclohexane,^53^ we constructed a cyclohexane oligomer (Figure 1c) to investigate its effects on the aggregation of the asphaltene models.

Although crude oil is a mixture of several hydrocarbon components, for the purpose of this study, the oil model was represented by only toluene. By definition, toluene is a good solvent for asphaltenes. It is chosen here because the aim is to test whether additives can promote asphaltene aggregates formation. In other words, we assume this system represents a starting point to quantify the increased aggregation of asphaltenes due to chemical additives.

### Simulation Systems

We constructed three simulation systems. Two of these (Systems 1 and 4) contain only toluene and asphaltene to benchmark the other simulations. The second group (Systems 2 and 5) and third group (Systems 3 and 6) of systems, as shown in Table 1, contain varying amounts of toluene and cyclohexane oligomer. In all systems, we used 12 asphaltene molecules to maintain the percentage by mass of asphaltene at ∼8 wt %,^13,16,54,55^ which is representative of asphaltene fraction in crude oil.^56,57^ Because of the difference in the molecular masses of the island and archipelago asphaltenes, different numbers of molecules of toluene and cyclohexane chain were inserted in our simulated systems. For example, since the archipelago model used has a slightly higher molecular mass, it requires more toluene and cyclohexane chains to maintain the three systems with comparable weight fractions.

**Table 1:** Composition of the Bulk and Kaolinite Systems Used in Simulations Conducted at 300 and 400 K^a^

system no.	asphaltene type	no. of asphaltene molecules	no. of toluene molecules	no. of cyclohexane chains	asphaltene weight fraction
1	ASPH	12	1000	0	8.5
2	ASPH	12	1000	22	7.8
3	ASPH	12	600	87	8.8
4	ARCH	12	1130	0	8.5
5	ARCH	12	1130	25	7.8
6	ARCH	12	670	100	8.8

a ASPH represents the island model asphaltene, while ARCH is the archipelago model. Only systems 1, 3, 4, and 6 were simulated at 400 K.

**Figure 2:**
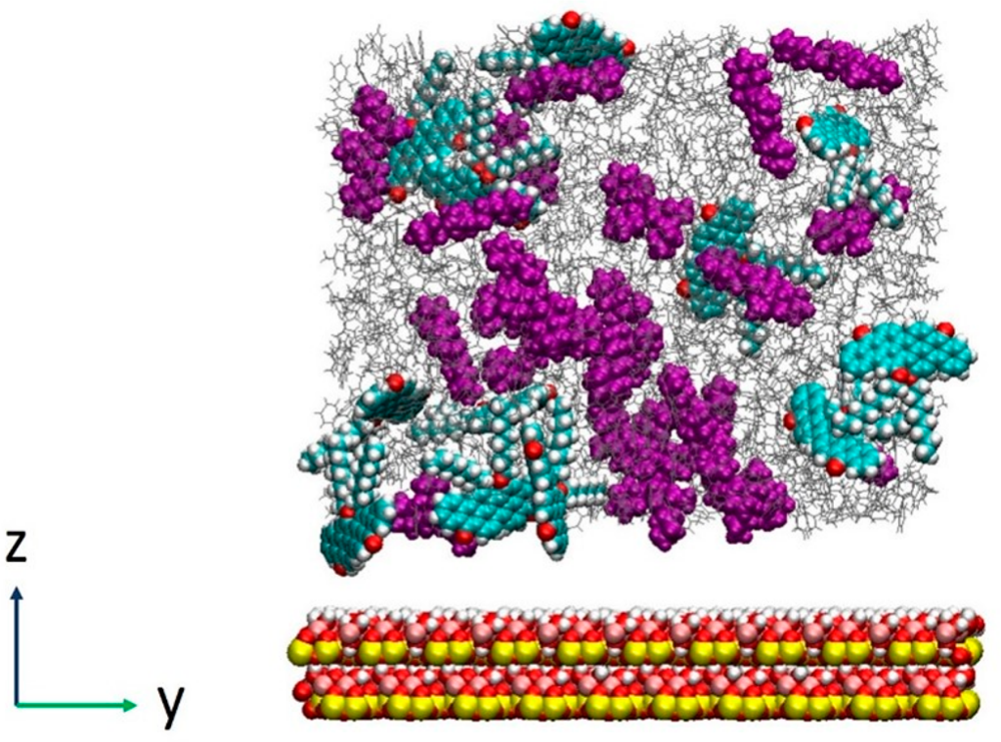
Representative snapshots of the initial configuration of the systems with hydrocarbons on the kaolinite surface. Cyclohexane chain = purple. Toluene = gray.

For all systems shown in Table 1, we conducted simulations in the bulk, and near a kaolinite surface. In the latter case, we constructed the simulation box by placing a thick slab of asphaltene, toluene, and cyclohexane oligomers on the kaolinite surface with dimensions 5.198 × 8.982 × 1.410 nm^3^ parallel to the *X*–*Y* plane, resulting in a simulation box of size 5.198 × 8.982 × 9.500 nm^3^. The asphaltene molecules were inserted randomly into the box, ensuring there is little aggregation at the start of the simulation. Figure 2 shows the snapshot of the initial configuration of the system. The hydrocarbon phase is sandwiched between two parallel solid surfaces, at a distance ranging between 3.7 and 4.5 nm. For simple fluids, confinement effects are pronounced within one or two molecular layers from the solid–liquid interface. The size of the system is chosen to be larger than that, but we cannot exclude a priori that confinement impacts the results. Unfortunately, at the resolution considered for this study, larger distances between the kaolinite surfaces quickly become prohibitive because of increased computational costs. The geometry chosen prevents the formation of a liquid–gas interface, which could compete for asphaltene adsorption compared to the solid–liquid interface.

The bulk simulation boxes were constructed in a similar manner, except without the kaolinite surface. The size of the simulation box used for bulk simulations was 5.198 × 8.982 × 6.500 nm^3^. The resultant density of the hydrocarbon was approximately 3.39 molecules/nm^3^. In all systems, periodic boundary conditions were applied in all directions in the simulation box.

### Force Fields

The Optimized Potentials for Liquid Simulations All Atom (OPLS-AA) force field^58^ was used to describe all hydrocarbons considered, following numerous related works in the literature.^13,14,56,59,60^ OPLS-AA has been shown to reproduce reasonably well experimental data for aromatic liquids.^61,62^ The hydroxylated kaolinite surfaces were modeled using the CLAYFF force field,^63^ following prior research on clay minerals from our group.^64,65^ The interfacial system of hydrocarbons interacting with clay surfaces has been studied previously by the combination of OPLS-AA and CLAYFF force fields.^66−68^ Prior studies confirmed that by combining CLAYFF and OPLS-AA force fields it is possible to achieve simulation results consistent with experimental observations. For example, Schampera et al.^69^ studied the interactions of organic cations on a montmorillonite clay surface, and their results were consistent with experimental XPS data. As another example, Ren and Liu^70^ and Phan et al.^71^ studied the structure of ethanol on alumina surfaces and found that the combination of these force fields compares well with experimental data.

Dispersive forces were modeled by the 12–6 Lennard-Jones (LJ) potential. Electrostatic forces were described by the Coulomb potential. The LJ parameters for unlike atomic interactions were obtained by using the geometric mixing rules, commonly used with the OPLS family of force fields. In all nonbonded interactions, the cut off radius for short-range interactions was set to 14 Å. Corrections for long-range electrostatic interactions were obtained by the particle mesh Ewald (PME) algorithm.^72,73^

### Algorithms

Atomistic MD simulations were conducted using the GROMACS package, version 5.1.4.^74,75^ After energy minimization, we carried out NVT canonical simulations for 1 ns to relax the initial configuration with the positions of the kaolinite surface kept fixed. The simulations were then conducted in an isobaric–isothermal (NPT) ensemble for 200 ns to ensure the densities and energies reached a stable state and enough for aggregation to be observed within a reasonable time scale. A time step of 1 fs was used for all simulations. During the NPT simulations, the kaolinite surface was restrained with a force constant of 1000 kJ/mol nm^2^ while asphaltenes, toluene, and the cyclohexane oligomer were allowed to move.

Subsequently, the time-averaged properties of the system (such as densities and radial distribution functions) were analyzed by conducting two additional 4 ns simulations for each system. The coordinates of the simulated systems were extracted every 0.5 ps to construct the simulation trajectories. The results were then quantified using the techniques described in the Results section. We set the temperature to either 300 or 400 K, with a relaxation time of 100 fs. The temperature was controlled by a Berendsen thermostat. The temperatures considered are representative of depths of up to approximately 2–3 km.^76−78^ To probe the possible mechanisms responsible for agglomeration, only Systems 1 and 3 (Table 1) were simulated at 400 K. The pressure was maintained at 15 MPa by the Berendsen barostat. This pressure represents that found in reservoirs.^36,79^ In the systems with a kaolinite surface, pressure coupling was applied only along the *Z* direction of the simulation box, perpendicular to the surface. This ensures that the surface area (*X* and *Y* dimensions) is constant for all systems.

## 3 Results

### Density Profiles

**Figure 3:**
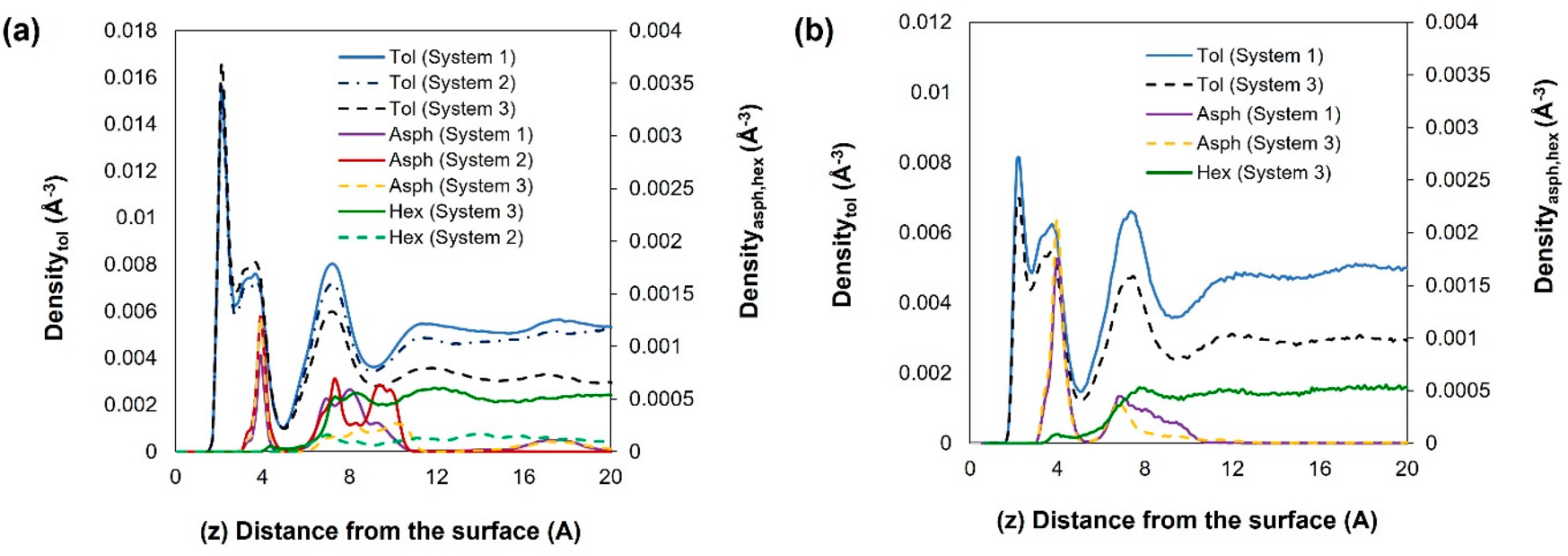
Centre of mass profiles along the *Z* direction, vertical from the kaolinite surface, for island asphaltene (ASPH), toluene (Tol), and cyclohexane oligomer (HEX) at (a) 300 and (b) 400 K. For system composition, we refer to Table 1.

The density profiles of the center of mass of toluene, island asphaltene (ASPH), and the cyclohexane oligomer (HEX), computed along the perpendicular (*Z*) direction at 300 and 400 K, are presented in Figure 3. The top plane of the hydroxyl ions represents the reference position for the vertical distance *Z*. From the results, we observe that at both temperatures, toluene adsorbs closest to the kaolinite surface, forming three layers with peaks at distances corresponding to 2.1, 3.7, and 7.3 Å from the surface. At 300 K, the first toluene layer achieves almost twice the density of the second and third layers. The island asphaltene adsorbs further from the surface from ∼3.1 Å up to 12 Å, with a pronounced peak at 3.9 Å, suggesting that toluene forms a “protective” layer on kaolinite. We can infer that toluene has stronger interactions with the kaolinite surface than the island asphaltene does. In contrast, the cyclohexane oligomer (HEX) yields no obvious layering on kaolinite. Our simulations show that adding HEX has no distinct effect on the structure of toluene and asphaltene. Increasing the temperature from 300 to 400 K leads to a reduction in the toluene density peaks and a slight increase in the first asphaltene density peak. This observation is consistent with recent AFM measurements conducted to study the adsorption of a model ASPH molecule, dissolved in toluene, and deposited on quartz. The AFM data showed that increasing temperature leads to a more direct adsorption of the poly aromatic hydrocarbon on the mineral surface.^35^

**Figure 4:**
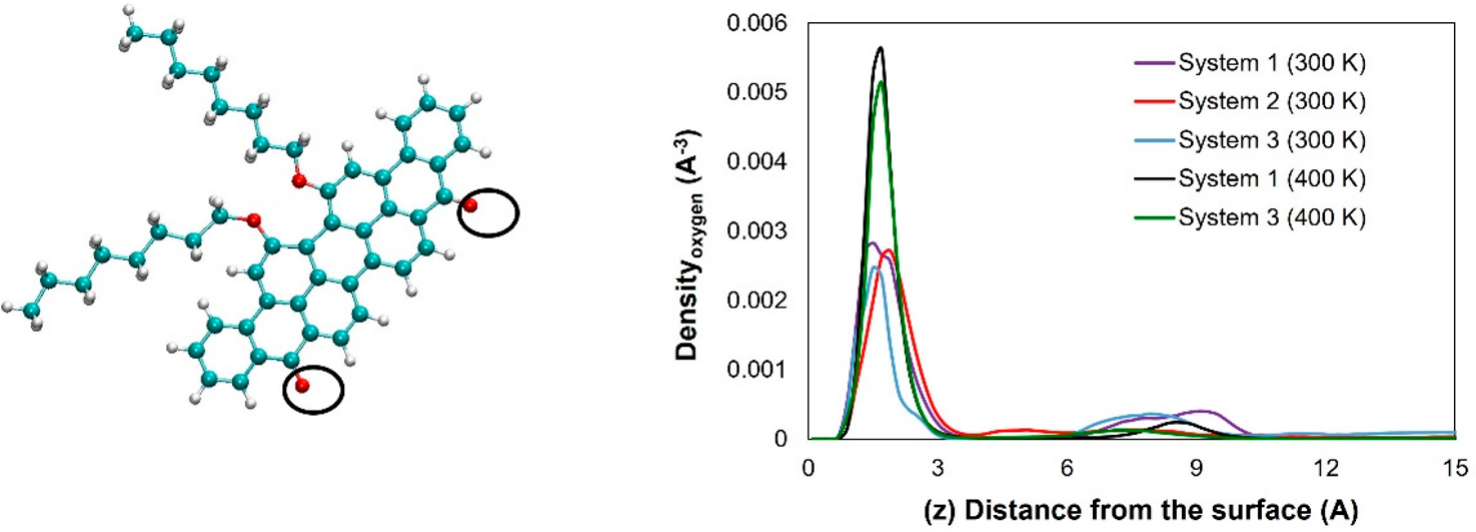
Atomic density profiles of two oxygen atoms (circled) located on island asphaltene (ASPH) along the *Z* direction, vertical from the kaolinite surface. Results are for simulations conducted at 300 and 400 K.

Although the center of mass of the asphaltenes seems further from the kaolinite surface than toluene, we expect that the presence of oxygen atoms could lead to bonds with the hydroxyl groups on the surface. To confirm this, we calculated the atomic density distribution of 2 oxygen atoms present in the asphaltene structure. The results are highlighted in Figure 4. We observe a sharp peak at ∼1.5–1.7 Å, which is closer to the surface than the density peaks computed for the centers of mass of both asphaltene and toluene. The height of the peak increases with temperature, in agreement with the observations from the center of mass density profiles of asphaltene. From the positions of these oxygen atoms, we infer that the main interactions of asphaltene with the surface are due to the presence of heteroatoms, which could potentially form hydrogen bonds with the hydroxyl ions on the mineral surface.

**Figure 5:**
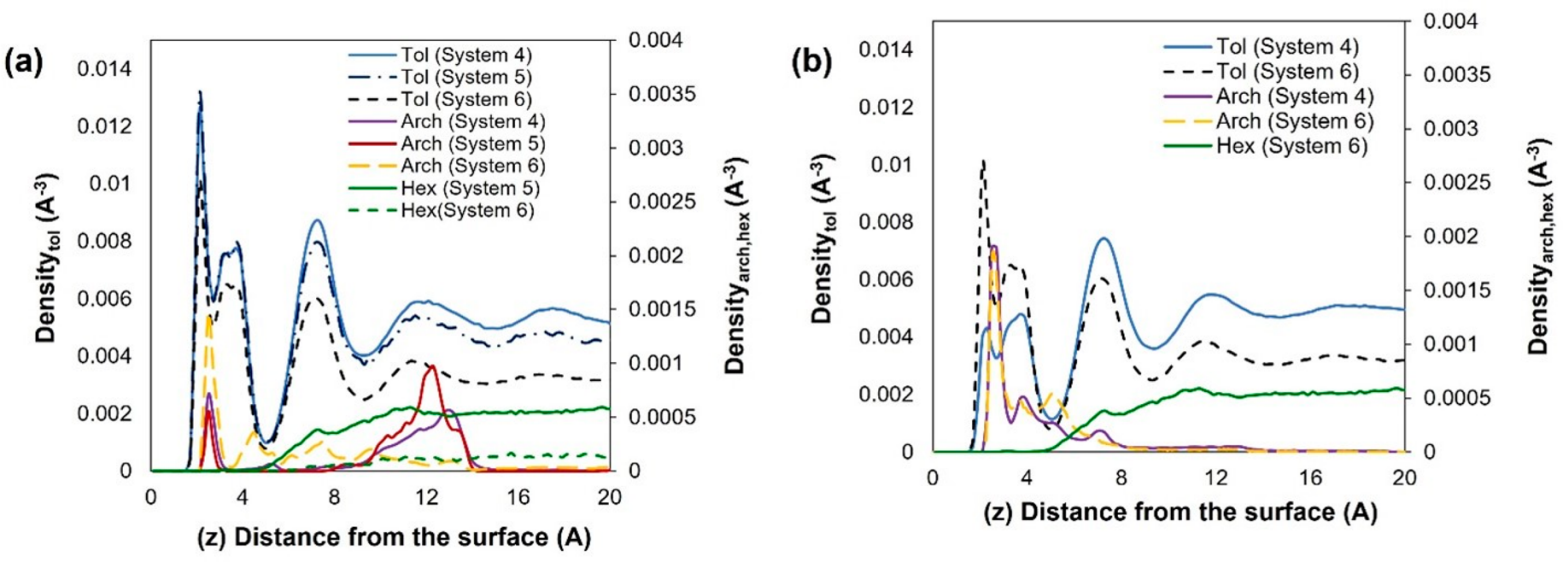
Centre of mass density profiles along the *Z* direction, vertical from the kaolinite surface, for the archipelago asphaltene (ARCH), toluene, and cyclohexane chain (HEX) at (a) 300 and (b) 400 K. System compositions are shown in Table 1.

In Figure 5, we provide the center of mass density profiles obtained for the systems containing the archipelago-structured asphaltene (ARCH). The positions of the toluene and HEX peaks correspond approximately to the positions shown in Figure 3. However, the archipelago-structured asphaltene adsorbs somewhat closer to kaolinite, with a distinct peak at 2.5 Å compared to the island asphaltene, whose first peak appears at 3.9 Å. A possible explanation is that the presence of sulfur, oxygen, and nitrogen atoms in the archipelago structure increases electrostatic and van der Waals interactions with the kaolinite surface (note in Figure 1 that the island model used here does not contain sulfur nor nitrogen atoms). At 300 K, a second asphaltene peak can be observed at 12.9 Å (System 4) and 12.1 Å (System 5); this peak disappears upon adding HEX or upon increasing the temperature to 400 K. This is due to the asphaltene packing at a closer distance to the surface, as observed from the noticeable increase in the height of the first asphaltene peak at 2.5 Å. It is worth noting that at 400 K, HEX does not significantly affect the density distribution of asphaltene near the surface.

**Figure 6:**
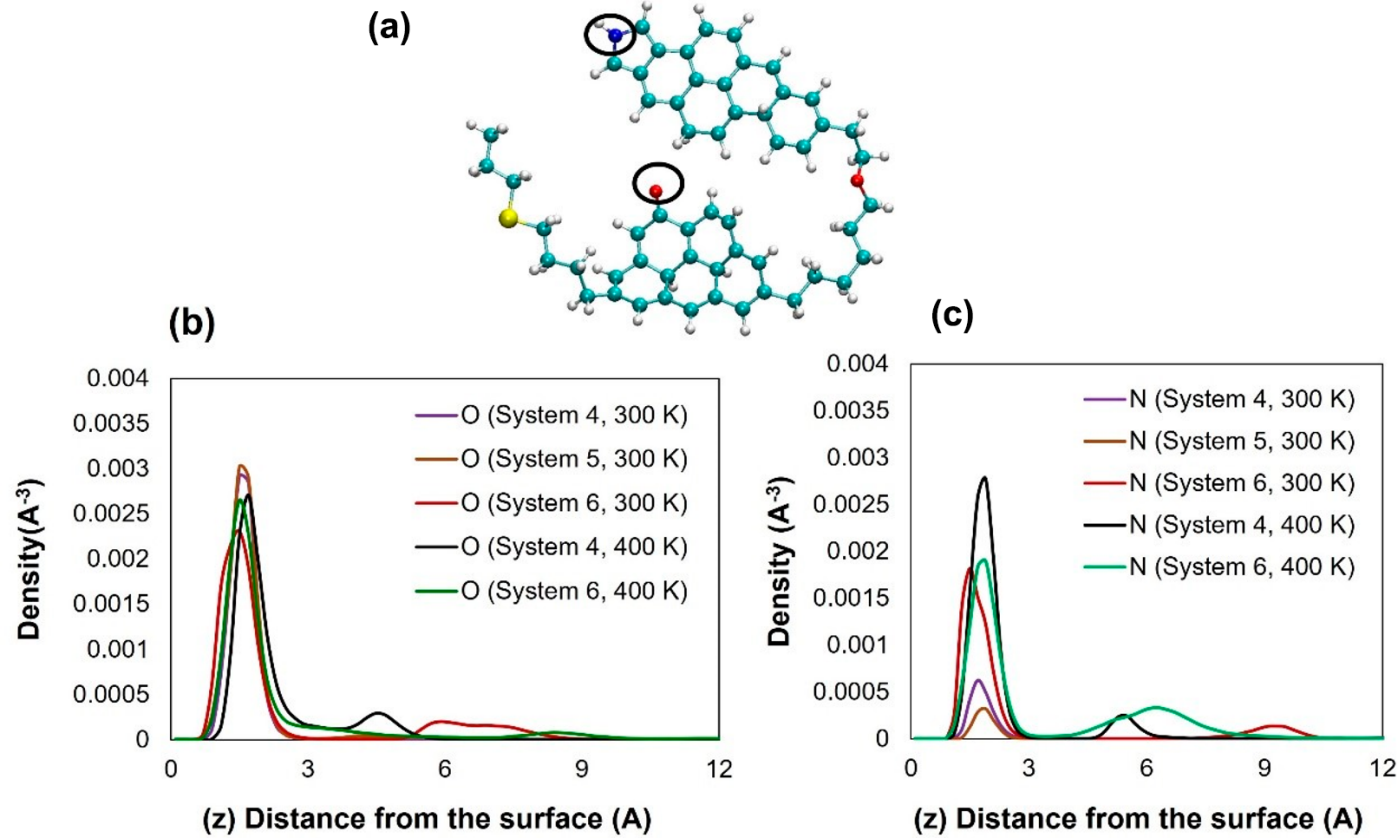
Atomic density profiles of oxygen atoms (panel b) and nitrogen atoms (panel c) found on ARCH along the *Z* direction, perpendicular to the kaolinite surface. Simulations results are obtained at 300 and 400 K. In panel a, the oxygen atom is highlighted by the red circled sphere, while nitrogen is highlighted by the blue circled sphere.

To probe the mechanisms responsible for the preferential adsorption, we computed the density profiles of the nitrogen and oxygen atoms found in the archipelago asphaltene structure. The results are presented in Figure 6, where it is shown that the relevant density peaks are found between 1.5 and 1.7 Å from kaolinite. These results highlight the interactions between the heteroatoms and clay mineral surfaces. It is worth noting that we did not observe a strong influence of HEX on the positions or intensity of the peaks, which could be due to the complex nature of the aggregation of archipelago asphaltenes.

### Cluster Size Analysis

**Figure 7:**
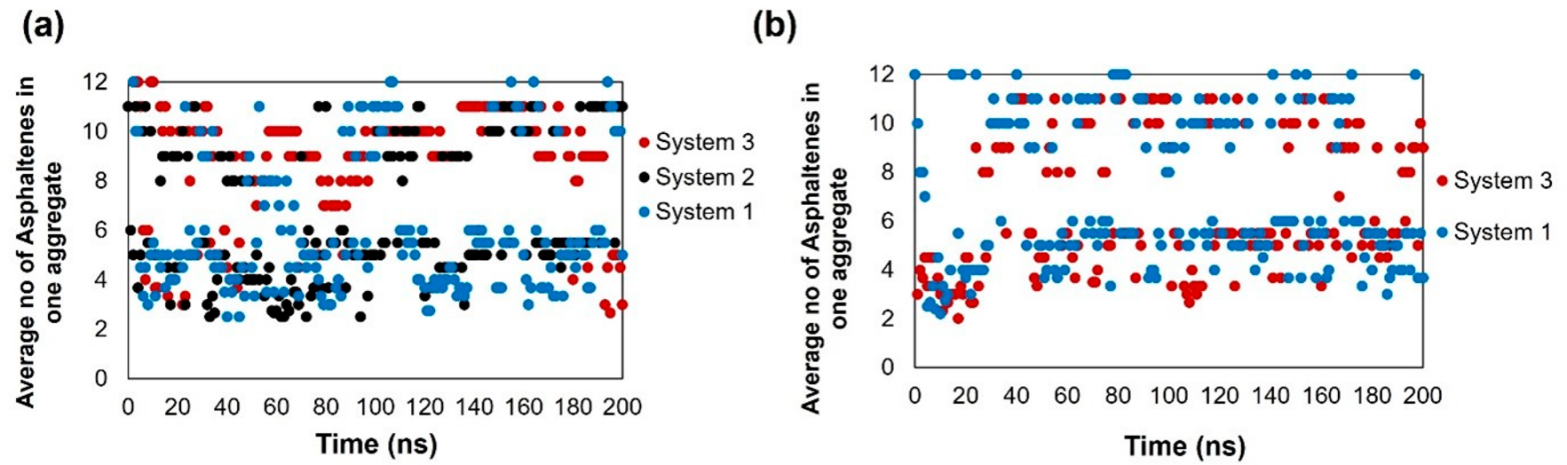
Average cluster (aggregate) as a function of time for the ASPH on the kaolinite surface at (a) 300 and (b) 400 K. System compositions are shown in Table 1.

To quantify the aggregation of asphaltene molecules, we estimated the average cluster size by calculating the number of asphaltene molecules in each aggregate and taking the average of the results obtained over the simulated trajectories. We also quantified the maximum number of asphaltenes in an aggregate. Following several studies in the literature,^13,18,30,38^ asphaltene molecules are considered clustered if the distance between them (distance of closest approach) is less than 3.5 Å. As shown in Figure 7a, at 300 K, the average cluster size of the island asphaltene (ASPH) increases as the number of HEX oligomers in the system increases. In the last 50 ns of the simulation, the average aggregate size in the system with only toluene (System 1) is ∼6–7. This increased to ∼7–8 and ∼8–9 in Systems 2 and 3, respectively. In System 3, asphaltenes yield one unstable aggregate, which breaks off repeatedly. The results in Figure S1, panel a, in the Supporting Information show that adding HEX leads to an increase in the maximum number of asphaltenes seen in any aggregate.

When the temperature is increased to 400 K, Figure 7b shows that there are more frequent fluctuations in the aggregate size, which is likely a consequence of the increase in the kinetic energy within the system. It is also interesting to note that the average size of the aggregates in systems 1 and 3 are ∼6.3 and ∼7.1, respectively. These results suggest that increasing the temperature increases the number of HEX required to cause a marked increase in the average cluster size, a result which is likely due to an increase in disorder of the system.

**Figure 8:**
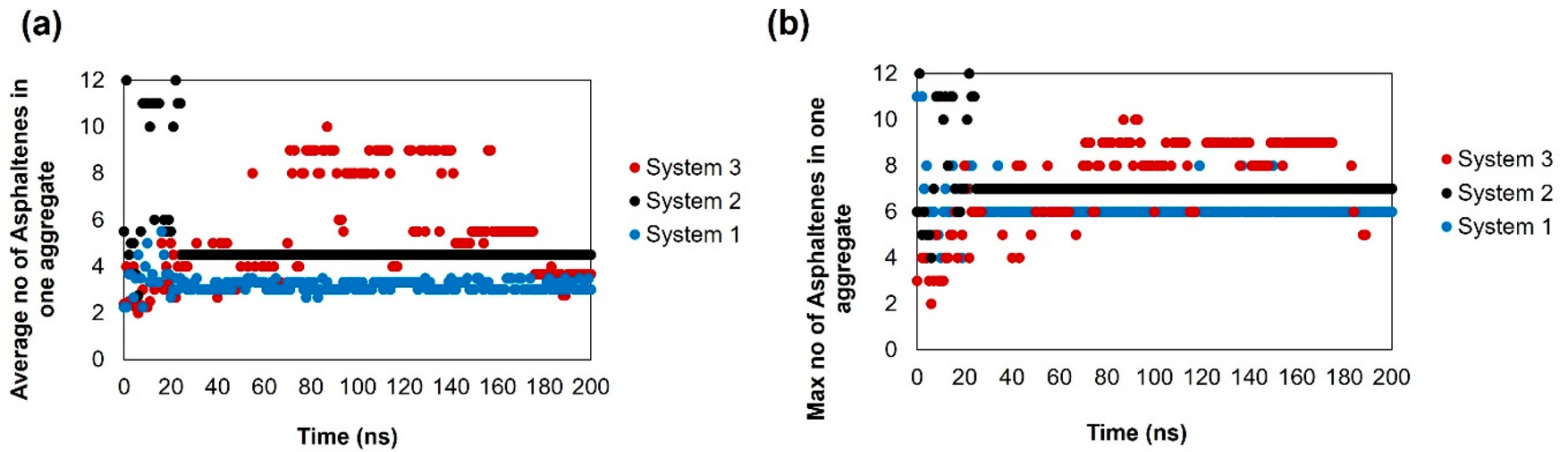
(a) Average cluster (aggregate) size and (b) maximum cluster size as a function of time for ASPH in the bulk systems at 300 K. System compositions are shown in Table 1.

We observe trends similar to those just discussed in the simulated bulk systems. In Figure 8, we present the cluster size of ASPH asphaltenes in the bulk. In System 1 (only toluene), the average size of the aggregates is ∼3–4, which increases to 4–5 with the addition of 22 HEX oligomers (System 2) and 5.8 with the presence of 87 HEX oligomers (System 3). In System 1, the maximum cluster size stabilizes at 6 asphaltenes, while this value increases to 7 and 8 in Systems 2 and 3, respectively. These results suggest that asphaltene aggregates in bulk systems are more stable and, on average, smaller than aggregates near kaolinite.

**Figure 9:**
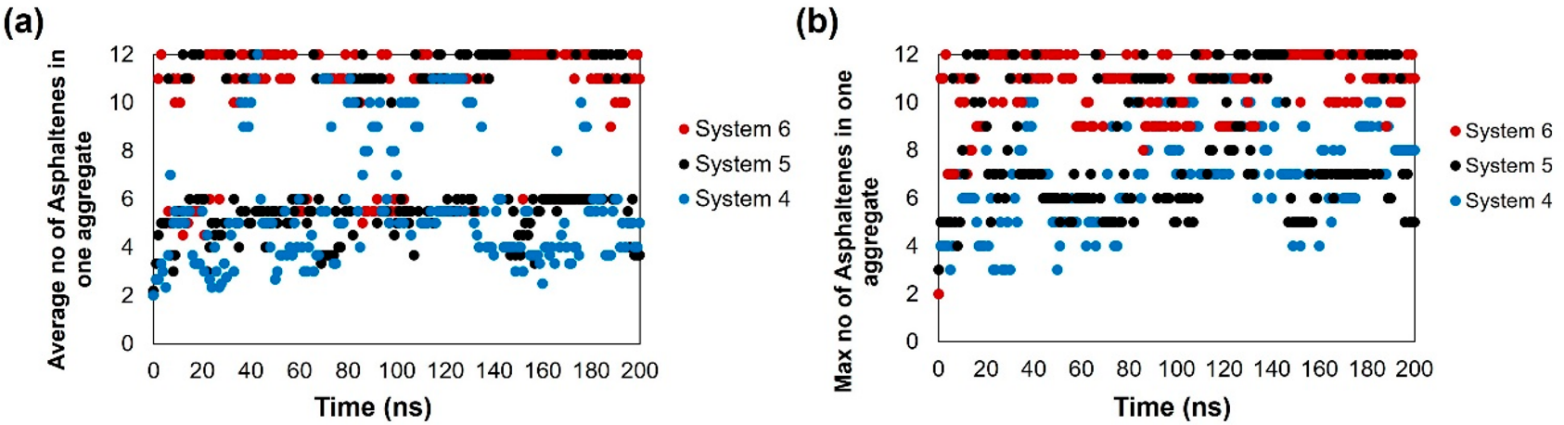
Average cluster (aggregate) size and (b) maximum cluster size as a function of time for ARCH on kaolinite at 300 K. System compositions are shown in Table 1.

In Figure 9, we provide cluster size analyses for archipelago asphaltene (ARCH) on kaolinite at 300 K. The effects due to the cyclohexane oligomer are similar to those discussed above for island asphaltene. In the last 50 ns, the average aggregate size increases from 6 to 10 when 100 HEX oligomers (System 6) replace some toluene in the system. Correspondingly, the maximum aggregate size increases from ∼7.9 to ∼10.9. The aggregates observed here are slightly larger than the aggregates formed by the island asphaltene.

In the bulk, the clustering results show that all the asphaltenes aggregate (Figure S2), even without HEX. The same is observed when the temperature is increased from 300 to 400 K in proximity of kaolinite. Snapshots of the fully aggregated asphaltenes are reported in the Supporting Information (Figure S3). We attribute this result to the difference in the size of the poly aromatic cores, length of the side aliphatic chains, and the presence of heteroatoms, compared to the relevant features of the island asphaltene structure. In the island asphaltene, the aromatic core contains 8 aromatic rings, whereas the archipelago asphaltene has 2 poly aromatic cores each of 5 and 6 aromatic rings, which could increase the π–π interactions.

### Radial Distribution Functions (RDF)

**Figure 10:**
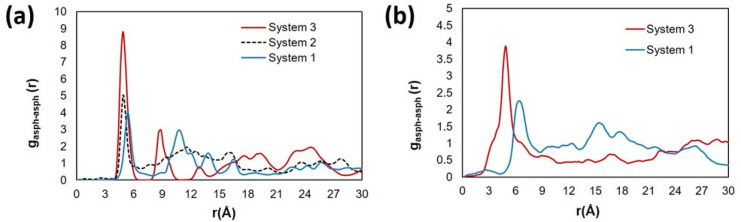
RDFs between the center of the aromatic cores of the island asphaltene (ASPH) pairs found in proximity of the kaolinite surface at (a) 300 and (b) 400 K. System compositions are shown in Table 1.

In Figure 10, we report the RDF between the center of mass of the aromatic cores of the island asphaltene (ASPH) at 300 and 400 K for the systems in proximity of the kaolinite surface. Because only layers occupied by asphaltenes (up to ∼1.5 nm), shown by the atomic density distributions, were considered for the RDF analyses, the systems are nearly planar. Because interfacial systems are not isotropic, the RDFs were computed in 2 dimensions. In system 1, panel a, the first most prominent peak is found at a radial distance of ∼5.3 Å. This suggests offset stacking of the aromatic cores or a slant orientation between asphaltene pairs. Carauta et al.^80^ reported from experiments that asphaltene dimers bind face–face at a distance of ∼3.6 Å in heptane and ∼5 Å in toluene. The peak distance is slightly greater than the π–π stacking distance of 5 Å. In the presence of HEX, the height of the first peak increases and is observed at a slightly closer radial distance of 4.8 Å, indicating stronger π–π interactions and a greater tendency to form aggregates. For all the systems, there are additional peaks observed at 10.6 Å (System 1), 11.3 Å (System 2), and 8.6 Å (System 3), suggesting the presence of multiple stacked structures.

The RDF at 400 K (panel b) shows a similar trend due to the HEX increasing the interactions between asphaltenes. In System 1, the most pronounced peak is found at a radial distance of ∼6.3 Å, which agrees with the possibility that at a higher temperature, the island asphaltene forms loose aggregates. However, in the presence of 87 HEX, the peak shifts to a smaller radial distance of ∼4.8 Å even at the higher temperature simulated. If we compare the RDF of the same system at two temperatures, we find that increasing the temperature reduces the probability of observing asphaltene cores close to each other.

**Figure 11:**
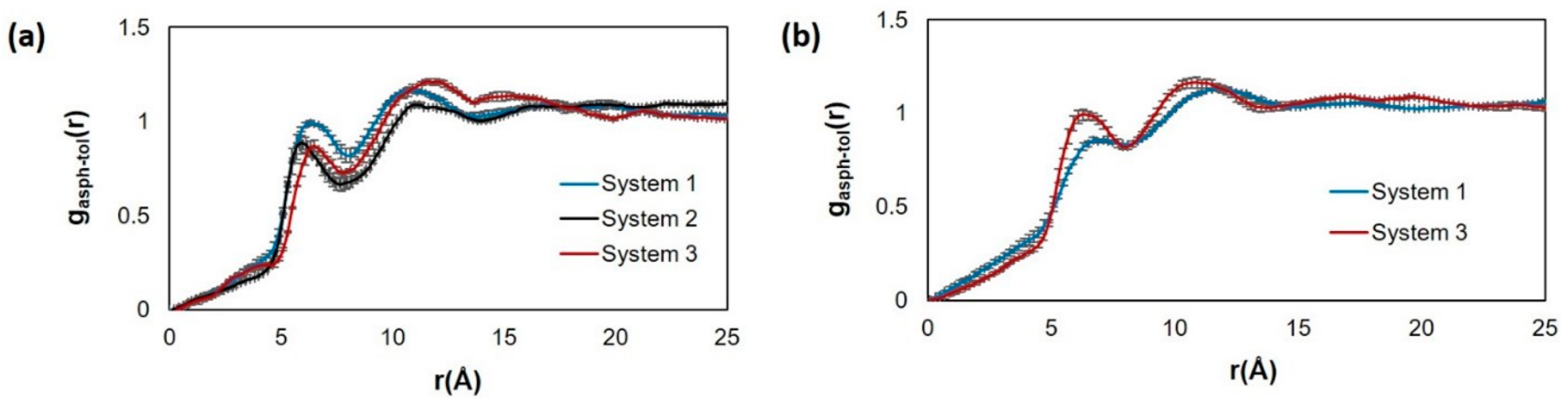
RDFs between the center mass of island asphaltene (ASPH) and the center of mass of toluene in proximity of kaolinite at (a) 300 and (b) 400 K. System compositions are shown in Table 1.

The toluene-ASPH RDF, presented in Figure 11, reveals the effects of HEX on the interactions between asphaltene and toluene. Interestingly, the results show that the presence of the HEX in the layers occupied by asphaltene has no strong effects on the structure of toluene around asphaltene at the two temperatures considered, even though in some systems, some HEX are present in the same layer as asphaltene and toluene. At both temperatures (panels a and b), the first and second peaks are located at ∼6.5 and 11.3 Å, respectively, although the height of the peak is slightly higher at the lower temperature (panel a).

For bulk systems containing the island asphaltene, Figure S4 of the Supporting Information demonstrates that the three-dimensional RDF for asphaltene pairs is similar to the results obtained for the systems simulated in proximity of kaolinite; HEX is also found to increase the height of the peak located at 4.8 Å. Moreover, the toluene-asphaltene three-dimensional RDF in the bulk is unaffected by the presence of HEX.

**Figure 12:**
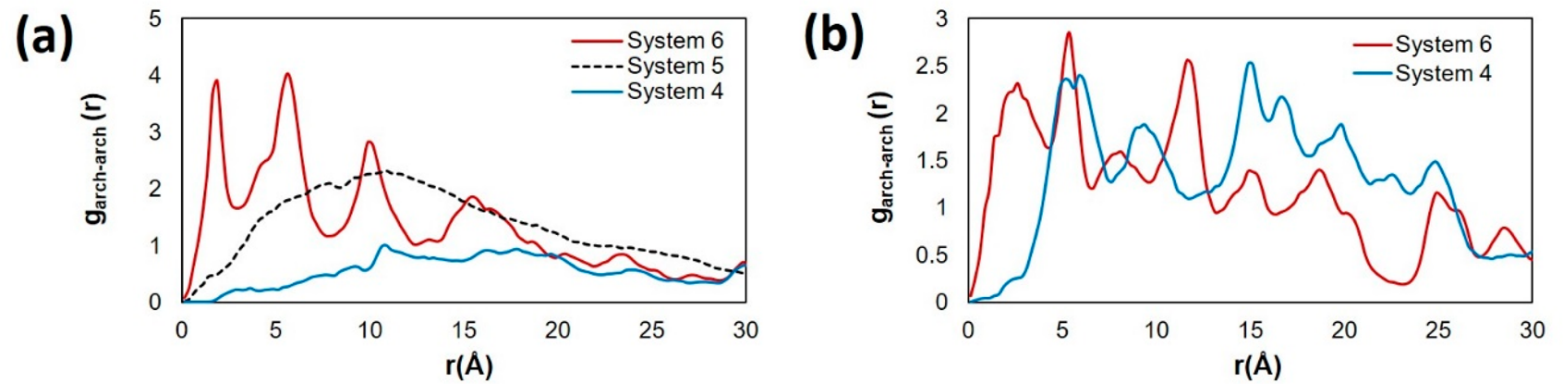
RDFs between the center of the archipelago asphaltene (ARCH) pairs at (a) and (b) 300 K and (c) 400 K. System compositions are shown in Table 1.

For the archipelago asphaltene (ARCH), we computed RDFs between the center of mass of the asphaltene pairs. Because the multiple aromatic cores and the heteroatoms present in the archipelago-like asphaltenes lead to more complex stacking and aggregation, we did not use the RDF between the aromatic cores to analyze stacking between asphaltene molecules. The results, in Figure 12, show that the presence of the cyclohexane chain leads to closer interactions between the archipelago asphaltene. In Systems 5 and 6 at 300 K (panel a), we observe multiple peaks, with the first peak found at ∼1.9 Å, whereas in system 4 with only toluene, there are no sharp peaks. This suggests that compact aggregates form in the presence of the cyclohexane chain. A similar trend is found at 400 K (panel b), where the first broad peak of system 4 is found at 5.1 Å, and upon the replacement with the cyclohexane oligomer, the peak shifts to a smaller distance of 2.8 Å. The asphaltene–toluene RDF, presented in Figure S5 in the Supporting Information, shows no visible differences between the system with only toluene and the systems with toluene and cyclohexane oligomer. In the RDFs at the two temperatures, there are no significant spikes, indicating that no well-defined aggregate forms between toluene and asphaltene molecules within the systems considered here.

### Orientation and Number of π–π Contacts of Island Asphaltene Molecules

**Figure 13:**
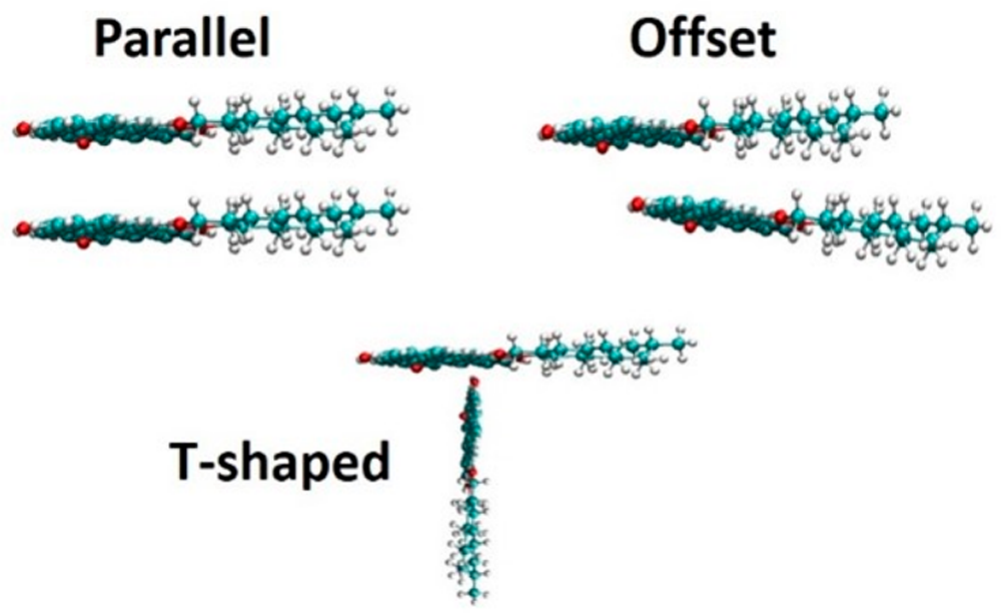
Possible stacking configurations of ASPH during aggregation.

To gain insights on the structure of the asphaltene aggregates, we calculated the angle formed between the poly aromatic planes of two asphaltenes in an aggregate on the kaolinite surface (illustrated in Figure 13). The angle ranges from 0 to 90° and could be face–face or T-shaped. In the face–face stacking, the center of aromatic cores could be aligned (parallel) or slightly displaced (offset). The orientation gives more information about the interactions between the aromatic cores of the asphaltene, with the parallel orientation being the strongest.

**Figure 14:**
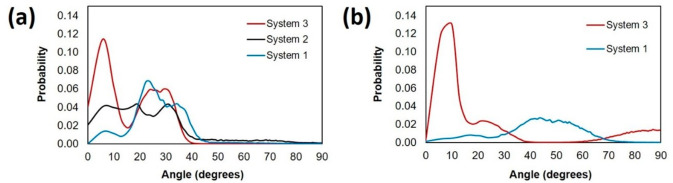
Angle between the poly aromatic planes of the island asphaltene (ASPH) pairs at (a) 300 and (b) 400 K on the kaolinite surface. System compositions are shown in Table 1.

In Figure 14, we report the angles between island asphaltene pairs at close contact (as identified by the first peaks in the RDFs of Figure 10) at 300 (panel a) and 400 K (panel b) for the kaolinite systems. As the number of HEX oligomers increases, the asphaltene pairs adopt a parallel orientation, suggesting a more compact aggregate structure established through π–π interactions. In System 1 at 300 K (panel a), there are two peaks at 5.7° and 23.7°. As the number of HEX increases, the probability of the angles between asphaltene pairs being less than 20° increases, with a pronounced peak at 5.7° in System 3. These results show that HEX oligomers shift the stacking between asphaltene from offset toward either parallel or offset structures. In the first RDF sphere of System 1 at 400 K (panel b), there is the highest probability of finding two asphaltene molecules oriented in a way to form angles between 40–50°, corresponding to a more T-shaped orientation. However, adding HEX shifts the peak to a smaller offset angle at 9.9°. The difference in the angles observed corroborates the findings that increasing temperature yields less compact aggregates. We observed similar trends in bulk systems (Figure S6).

**Figure 15:**
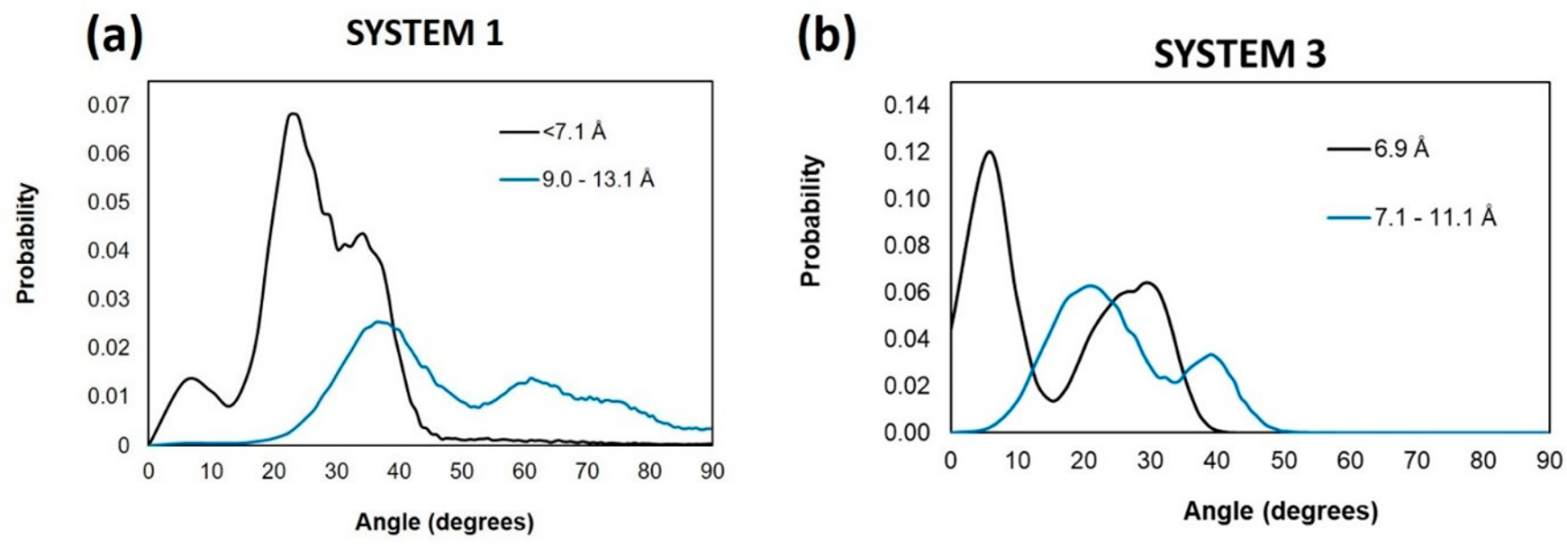
Angle between the poly aromatic planes of the ASPH pairs versus distance between them in (a) System 1 and (b) System 3 on kaolinite surface. System compositions are shown in Table 1.

We also quantified the relationship between the distance between asphaltene pairs and the type of stacking (Figure 15). We observed that as the distance between the asphaltenes increases, the orientation tends toward offset or T-shaped. In System 1, asphaltene molecules found at distances less than 7.1 Å are mostly offset, whereas those found at larger distances show a preference for T-shaped orientations.

To complement this analysis, we quantified the number of π–π contacts between aromatic cores of the island asphaltenes. A contact was considered established when the distance between the center of mass of the aromatic cores is less than ∼4.8 Å (the radial distance at which peaks are observed on the RDF). The results in Figure S7 in the Supporting Information show that the HEX oligomers increase the number of compact π–π contacts in the aggregated asphaltenes. In the presence of the kaolinite surface, at 300 K (panel a), the number of contacts in the absence of HEX fluctuates between 0 and 1; this value increases to 2–4 in System 3. At 400 K (panel b), the number of contacts decreases, in agreement with our previous observations that increasing the temperature leads to less compact aggregates. In panel c, we find that the number of π–π contacts is higher in the bulk phase than in the presence of the kaolinite slab. We see in panel c that the number of contacts in System 1 is between 0 and 2, while in System 3, the value increases to 6, indicating more rigid aggregates in bulk systems compared to the systems near kaolinite.

### Compactness of Archipelago Asphaltenes

**Figure 16:**
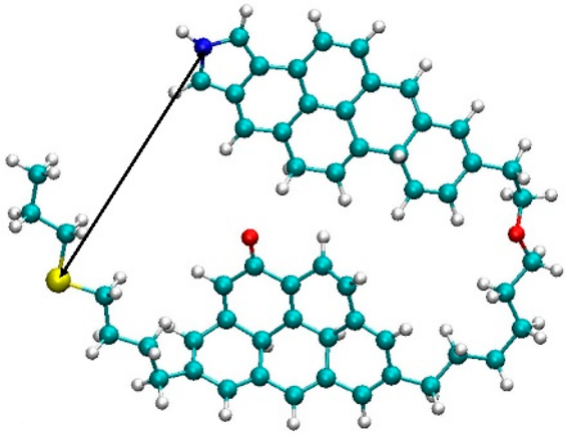
Illustration of the distance between sulfur and nitrogen atoms calculated for ARCH molecules.

**Figure 17:**
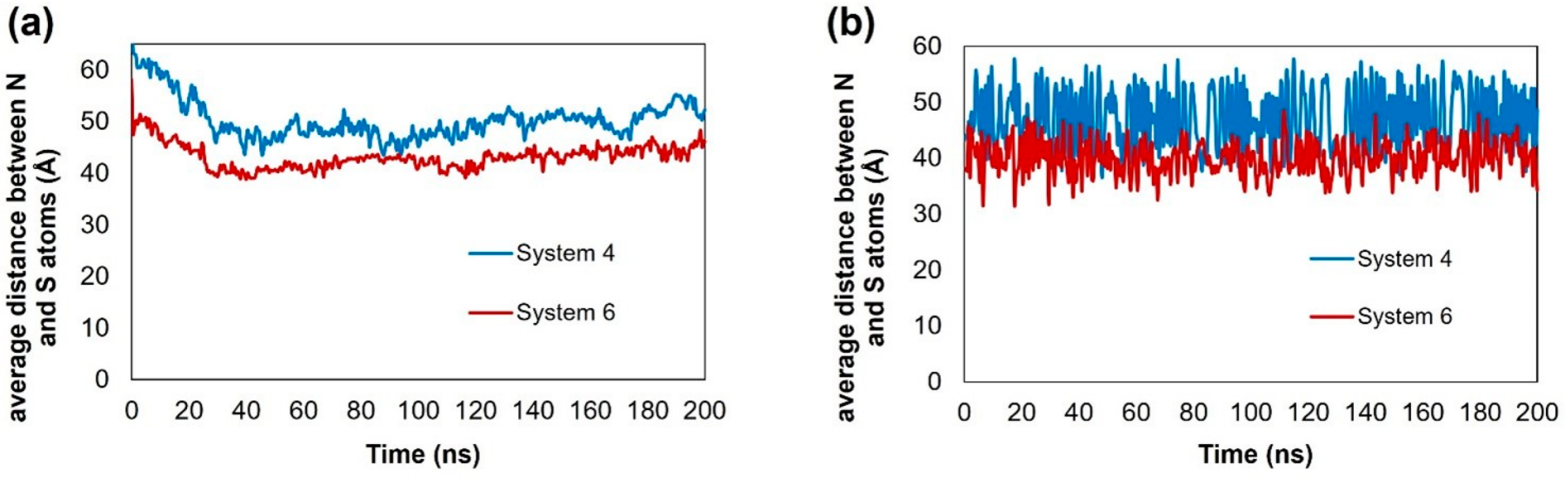
Distance between sulfur and nitrogen atoms calculated for ARCH (a) on the kaolinite surface and (b) in the bulk at 300 K. System compositions are shown in Table 1.

The asphaltenes of the archipelago type show more complex aggregation because of their extended molecular structure. To evaluate their structure, we quantified the distance between nitrogen (N) and sulfur (S) atoms in individual asphaltenes (see Figure 16). The results reported in Figure 17 (panel a) show that in the proximity of kaolinite at 300 K, although the asphaltene seems to completely unfold, the distance between the N and S atoms reduces slightly from ∼51 Å in System 4 to ∼43 Å in System 6, yielding slightly more compact molecules. Increasing the temperature to 400 K (Figure S8) reduces these values to ∼34 Å, which is consistent with high degree of aggregation, for both systems considered. In contrast to the effects of temperature on the island asphaltenes, increasing temperature makes the archipelago asphaltenes more compact. The corresponding results for bulk systems are presented in Figure 17 (panel b). HEX leads to a reduction in the distance, but it is worth noting that the effect is not very pronounced near kaolinite.

We also computed the radius of gyration (*R*_g_) to support the analysis above. In the bulk phase (Figure S9), *R*_g_ fluctuates within a wider range than in proximity of kaolinite (Figure S10). On kaolinite at 300 K (Figure S10a), *R*_g_ reduces from ∼38 Å in System 4 to ∼31 Å in System 6 upon adding HEX oligomers. At 400 K (Figure S10b), *R*_g_ remains at ∼35 Å in both systems.

### Interaction Energies and Entropy

In Table 2, we report van der Waals (Δ*E*_VDW_) and electrostatic interaction potentials (Δ*E*_ELEC_) for ASPH molecules in the systems studied. The interactions between asphaltenes are dominated slightly (∼55%) by electrostatic interactions, due to the presence of heteroatoms, which increase molecular polarity. The presence of HEX oligomers has greater effect on the van der Waal interactions, which complements the observations of increase in π–π interactions. The interactions of the archipelago - structured asphaltenes vary in a similar manner and are shown in Table S1 in the SI.

**Table 2:** Asphaltene–Asphaltene Interactions between Island Asphaltenes (ASPH) on the Kaolinite Surface and in Bulk Systems^a^

	Kaolinite at 300 K		Kaolinite at 400 K		Bulk at 300 K	
System	Asp-Asp(Δ*E*_VDW_)/kJ mol^–1^	Asp-Asp(Δ*E*_ELEC_)/kJ mol^–1^	Asp-Asp(Δ*E*_VDW_)/kJ mol^–1^	Asp-Asp(Δ*E*_ELEC_)/kJ mol^–1^	Asp-Asp(Δ*E*_VDW_)/kJ mol^–1^	Asp-Asp(Δ*E*_ELEC_)/kJ mol^–1^
1	–834 ± 3	–1053 ± 4	–770 ± 2	–1086 ± 10	–825 ± 21	–1075 ± 27
2	–871 ± 2	–1060 ± 1	–	–	–880 ± 15	–1047 ± 25
3	–1128 ± 55	–1047 ± 3	–891 ± 1	–1088 ± 3	–945 ± 1	–1082 ± 15

a Only short-range contributions to potential energies are considered. System compositions are shown in Table 1.

The configurational entropy of asphaltene in toluene in the presence of HEX oligomers was estimated by applying the method described by Schlitter.^81^ We determined the entropy of 3 asphaltenes with similar mass percentage to those used in our systems. Even though the uncertainty is large as a consequence of the small number of molecules considered for this calculation, the results in Table 3 suggest that the presence of HEX oligomers reduces the entropy of the asphaltenes, indicative of a more ordered structure. As expected, increasing the temperature leads to more disordered systems (higher entropy), which is consistent with results discussed above.

**Table 3:** Configurational Entropy of 3 Asphaltenes in Bulk Systems at 300 and 400 K^a^

System (TΔ*S*)/kJmol^–1^	Island	Archipelago
System1/4 at 300 K	803 ± 3	811 ± 4
System3/6 at 300 K	798 ± 3	792 ± 6
System1/4 at 400 K	1129 ± 1	1190 ± 4
System3/6 at 400 K	1114 ± 5	1178 ± 10

a System compositions are shown in Table S2.

## 4 Summary

We performed molecular dynamics simulations to study the aggregation of asphaltene, a major component of crude oils, in the proximity of a kaolinite surface and in the bulk. As a proof of concept, to determine the effects of a cyclohexane oligomer on model island and archipelago asphaltenes, we analyzed the aggregation of asphaltene in toluene, in the presence or absence of the oligomer, at 300 and 400 K temperatures, at the pressure of 15 MPa. The results suggest that the cyclohexane oligomer slightly increases the level of aggregation of asphaltene. In the island asphaltene, this was evident by the increase in the number of parallel π–π orientations of the aggregates, whereas in the archipelago structure, we observed more compact asphaltenes.

We found that the aggregation of the island model was more pronounced near kaolinite compared to the aggregation in the bulk phase, while the aggregation of the archipelago model was less pronounced. It is possible that the difference in the interactions between asphaltene molecules and the kaolinite surface, mediated with the formation of a dense toluene layer near the solid surface, contributes to this result. Our energetic and entropic analyses align with our observations. It should, however, be noted that confinement effects cannot be ruled out, given the relatively small distance between the two kaolinite surfaces confining the system investigated.

Overall, our study contributes to our understanding of the aggregation of asphaltenes near the kaolinite clay surface and the influence of the structure on aggregation behavior. For future work, larger systems with more asphaltene models should be investigated to monitor in greater detail the stages of aggregation that we could not implement via MD simulations due to high computational costs. A suitable alternative could be large-scale, coarse-grained molecular simulations. Experiments such as dynamic light scattering could also be conducted to directly investigate the aggregation of asphaltenes and compare the results to simulation results.^82^

## Supporting Information Available

Maximum cluster size of island asphaltenes as a function of time; average cluster size as a function of time for the archipelago asphaltene on kaolinite at 400 K and in bulk systems at 300 K; RDFs between center mass of island asphaltene and center of mass of asphaltene and toluene in bulk systems at 300 K; RDFs between the center mass of archipelago asphaltene and the center of mass of toluene on kaolinite surface; angle between the poly aromatic planes of the island asphaltene pairs; number of π–π contacts between the aromatic cores of island asphaltene pairs on kaolinite; distance between sulfur and nitrogen atoms for the archipelago asphaltene on kaolinite at 400 K; radius of gyration of individual archipelago asphaltene in kaolinite and bulk systems; asphaltene-asphaltene interaction energies between archipelago asphaltenes; representative simulation snapshots (PDF)

## References

[ref1] Syunyaev R. Z.; Balabin R. M.; Akhatov I. S.; Safieva J. O. Adsorption of Petroleum Asphaltenes onto Reservoir Rock Sands Studied by Near-Infrared (NIR) Spectroscopy. Energy Fuels 2009, 23, 1230–6. 10.1021/ef8006068.

[ref2] Mullins O. C. The Modified Yen Model. Energy Fuels 2010, 24, 2179–207. 10.1021/ef900975e.

[ref3] Jafari Behbahani T.; Ghotbi C.; Taghikhani V.; Shahrabadi A. Asphaltene Deposition under Dynamic Conditions in Porous Media: Theoretical and Experimental Investigation. Energy Fuels 2013, 27, 622–39. 10.1021/ef3017255.

[ref4] Yaseen S.; Mansoori G. A. Molecular Dynamics Studies of Interaction between Asphaltenes and Solvents. J. Pet Sci. Eng. 2017, 156, 118–24. 10.1016/j.petrol.2017.05.018.

[ref5] Verdier S.; Carrier H.; Andersen S. I.; Daridon J.-L. Study of Pressure and Temperature Effects on Asphaltene Stability in Presence of CO2. Energy Fuels 2006, 20, 1584–90. 10.1021/ef050430g.

[ref6] Rogel E.; Ovalles C.; Moir M. Asphaltene Stability in Crude Oils and Petroleum Materials by Solubility Profile Analysis. Energy Fuels 2010, 24, 4369–74. 10.1021/ef100478y.

[ref7] Mullins O. C. The Asphaltenes. Annu. Rev. Anal Chem. 2011, 4, 393–418. 10.1146/annurev-anchem-061010-113849. 21689047

[ref8] Korb J.-P.; Louis-Joseph A.; Benamsili L. Probing Structure and Dynamics of Bulk and Confined Crude Oils by Multiscale NMR Spectroscopy, Diffusometry, and Relaxometry. J. Phys. Chem. B 2013, 117, 7002–14. 10.1021/jp311910t. 23687962

[ref9] Sedghi M.; Goual L.; Welch W.; Kubelka J. Effect of Asphaltene Structure on Association and Aggregation Using Molecular Dynamics. J. Phys. Chem. B 2013, 117, 5765–76. 10.1021/jp401584u. 23581711

[ref10] Silva H. S.; Sodero A. C. R.; Bouyssiere B.; Carrier H.; Korb J.-P.; Alfarra A.; Vallverdu G.; Bégué D.; Baraille I. Molecular Dynamics Study of Nanoaggregation in Asphaltene Mixtures: Effects of the N, O, and S Heteroatoms. Energy Fuels 2016, 30, 5656–64. 10.1021/acs.energyfuels.6b01170.

[ref11] Mullins O. C.; Sabbah H.; Eyssautier J.; Pomerantz A. E.; Barré L.; Andrews A. B.; Ruiz-Morales Y.; Mostowfi F.; McFarlane R.; Goual L.; Lepkowicz R.; Cooper T.; Orbulescu J.; Leblanc R. M.; Edwards J.; Zare R. N. Advances in Asphaltene Science and the Yen-Mullins Model. Energy Fuels 2012, 26, 3986–4003. 10.1021/ef300185p.

[ref12] Santos Silva H.; Alfarra A.; Vallverdu G.; Bégué D.; Bouyssiere B.; Baraille I. Sensitivity of Asphaltene Aggregation toward the Molecular Architecture under Desalting Thermodynamic Conditions. Energy Fuels 2018, 32, 2681–92. 10.1021/acs.energyfuels.7b02728.

[ref13] Headen T. F.; Boek E. S.; Jackson G.; Totton T. S.; Müller E. A. Simulation of Asphaltene Aggregation through Molecular Dynamics: Insights and Limitations. Energy Fuels 2017, 31, 1108–25. 10.1021/acs.energyfuels.6b02161.

[ref14] Zi M.; Chen D.; Ji H.; Wu G. Effects of Asphaltenes on the Formation and Decomposition of Methane Hydrate: A Molecular Dynamics Study. Energy Fuels 2016, 30, 5643–50. 10.1021/acs.energyfuels.6b01040.

[ref15] Wang W.; Taylor C.; Hu H.; Humphries K. L.; Jaini A.; Kitimet M.; Scott T.; Stewart Z.; Ulep K. J.; Houck S.; Luxon A.; Zhang B.; Miller B.; Parish C. A.; Pomerantz A. E.; Mullins O. C.; Zare R. N. Nanoaggregates of Diverse Asphaltenes by Mass Spectrometry and Molecular Dynamics. Energy Fuels 2017, 31, 9140–51. 10.1021/acs.energyfuels.7b01420.

[ref16] Boek E. S.; Yakovlev D. S.; Headen T. F. Quantitative Molecular Representation of Asphaltenes and Molecular Dynamics Simulation of Their Aggregation. Energy Fuels 2009, 23, 1209–19. 10.1021/ef800876b.

[ref17] Pacheco-Sánchez J. H.; Álvarez-Ramírez F.; Martínez-Magadán J. M. Morphology of Aggregated Asphaltene Structural Models. Energy Fuels 2004, 18, 1676–86. 10.1021/ef049911a.

[ref18] Mohammed S.; Gadikota G. The Role of Calcite and Silica Interfaces on the Aggregation and Transport of Asphaltenes in Confinement. J. Mol. Liq. 2019, 274, 792–800. 10.1016/j.molliq.2018.10.163.

[ref19] Alvim R. S.; Lima F. C. D. A.; Sánchez V. M.; Headen T. F.; Boek E. S.; Miranda C. R. Adsorption of asphaltenes on the calcite (10.4) surface by first-principles calculations. RSC Adv. 2016, 6, 95328–36. 10.1039/C6RA19307B.

[ref20] Liu F.; Yang H.; Wang J.; Qian Y.; Wu J.; Li S.; Liu Q.; Yang S.; Xu S.; Zhang X.; Zhao Z.; Wang J. Molecular Interaction between Asphaltene and Quartz with Different Surface Wettability: A Combined Study of Experimental Measurement and Theoretical Calculation. Fuel 2019, 258, 115937 10.1016/j.fuel.2019.115937.

[ref21] Headen T. F.; Boek E. S. Potential of Mean Force Calculation from Molecular Dynamics Simulation of Asphaltene Molecules on a Calcite Surface. Energy Fuels 2011, 25, 499–502. 10.1021/ef1010385.

[ref22] Saraji S.; Goual L.; Piri M. Dynamic Adsorption of Asphaltenes on Quartz and Calcite packs in the Presence of Brine Films. Colloids Surf: A Physicochem Eng. Asp 2013, 434, 260–7. 10.1016/j.colsurfa.2013.05.070.

[ref23] Natarajan A.; Kuznicki N.; Harbottle D.; Masliyah J.; Zeng H.; Xu Z. Understanding Mechanisms of Asphaltene Adsorption from Organic Solvent on Mica. Langmuir 2014, 30, 9370–7. 10.1021/la500864h. 24978299

[ref24] Nassar N. N.; Hassan A.; Pereira-Almao P. Effect of Surface Acidity and Basicity of Aluminas on Asphaltene Adsorption and Oxidation. J. Colloid Interface Sci. 2011, 360, 233–8. 10.1016/j.jcis.2011.04.056. 21571295

[ref25] Lu N.; Dong X.; Chen Z.; Liu H.; Zheng W.; Zhang B. Effect of Solvent on the Adsorption Behavior of Asphaltene on Silica Surface: A Molecular Dynamic Simulation Study. J. Pet Sci. Eng. 2022, 212, 110212 10.1016/j.petrol.2022.110212.

[ref26] Lan T.; Zeng H.; Tang T. Understanding Adsorption of Violanthrone-79 as a Model Asphaltene Compound on Quartz Surface Using Molecular Dynamics Simulations. J. Phys. Chem. C 2018, 122, 28787–96. 10.1021/acs.jpcc.8b09712.

[ref27] Liu F.; Yang H.; Yang M.; Wu J.; Yang S.; Yu D.; Wu X.; Wang J.; Gates I.; Wang J. Effects of Molecular Polarity on the Adsorption and Desorption Behavior of Asphaltene Model Compounds on Silica Surfaces. Fuel 2021, 284, 118990 10.1016/j.fuel.2020.118990.

[ref28] Xiong Y.; Cao T.; Chen Q.; Li Z.; Yang Y.; Xu S.; Yuan S.; Sjöblom J.; Xu Z. Adsorption of a Polyaromatic Compound on Silica Surfaces from Organic Solvents Studied by Molecular Dynamics Simulation and AFM Imaging. J. Phys. Chem. C 2017, 121, 5020–8. 10.1021/acs.jpcc.6b11763.

[ref29] Li X.; Bai Y.; Sui H.; He L. Understanding the Liberation of Asphaltenes on the Muscovite Surface. Energy Fuels 2017, 31, 1174–81. 10.1021/acs.energyfuels.6b02278.

[ref30] Mohammed S.; Gadikota G. The Influence of CO2 on the Structure of Confined Asphaltenes in Calcite Nanopores. Fuel 2019, 236, 769–77. 10.1016/j.fuel.2018.08.124.

[ref31] Fang T.; Wang M.; Li J.; Liu B.; Shen Y.; Yan Y.; Zhang J. Study on the Asphaltene Precipitation in CO2 Flooding: a Perspective from Molecular Dynamics Simulation. Ind. Eng. Chem. Res. 2018, 57, 1071–7. 10.1021/acs.iecr.7b03700.

[ref32] Li X.; Chi P.; Guo X.; Sun Q. CO2-Induced Asphaltene Deposition and Wettability Alteration on a Pore Interior Surface. Fuel 2019, 254, 115595 10.1016/j.fuel.2019.06.003.

[ref33] Li Z.; Gong K.; Wang J.; Hao Y.; Yan Y.; Zhang J. Molecular Insights into Asphaltene Aggregation in Gas Flooding. Energy Fuels 2022, 36, 762–70. 10.1021/acs.energyfuels.1c03004.

[ref34] Jian C.; Poopari M. R.; Liu Q.; Zerpa N.; Zeng H.; Tang T. Reduction of Water/Oil Interfacial Tension by Model Asphaltenes: The Governing Role of Surface Concentration. J. Phys. Chem. B 2016, 120, 5646–54. 10.1021/acs.jpcb.6b03691. 27268710

[ref35] Lan T.; Liu J.; Zeng H.; Tang T. Temperature-Induced Transition from Indirect to Direct Adsorption of Polycyclic Aromatic Hydrocarbons on Quartz: A Combined Theoretical and Experimental Study. Ind. Eng. Chem. Res. 2020, 59, 18500–9. 10.1021/acs.iecr.0c02412.

[ref36] Headen T. F.; Boek E. S. Molecular Dynamics Simulations of Asphaltene Aggregation in Supercritical Carbon Dioxide with and without Limonene. Energy Fuels 2011, 25, 503–8. 10.1021/ef1010397.

[ref37] Aminzadeh R.; Nikazar M.; Dabir B. The Effect of Nonylphenol on Asphaltene Aggregation: A Molecular Dynamics Approach. Pet Sci. Technol. 2019, 37, 1883–90. 10.1080/10916466.2017.1305402.

[ref38] Ghamartale A.; Zendehboudi S.; Rezaei N. New Molecular Insights into Aggregation of Pure and Mixed Asphaltenes in the Presence of N-Octylphenol Inhibitor. Energy Fuels 2020, 34, 13186–207. 10.1021/acs.energyfuels.0c02443.

[ref39] Goual L.; Sedghi M.; Wang X.; Zhu Z. Asphaltene Aggregation and Impact of Alkylphenols. Langmuir 2014, 30, 5394–403. 10.1021/la500615k. 24784502

[ref40] Lin Y.-J.; He P.; Tavakkoli M.; Mathew N. T.; Fatt Y. Y.; Chai J. C.; Goharzadeh A.; Vargas F. M.; Biswal S. L. Characterizing Asphaltene Deposition in the Presence of Chemical Dispersants in Porous Media Micromodels. Energy Fuels 2017, 31, 11660–8. 10.1021/acs.energyfuels.7b01827.

[ref41] Jiang B.; Zhang R.; Yang N.; Zhang L.; Sun Y.; Jian C.; Liu L.; Xu Z. Molecular Mechanisms of Suppressing Asphaltene Aggregation and Flocculation by Dodecylbenzenesulfonic Acid Probed by Molecular Dynamics Simulations. Energy Fuels 2019, 33, 5067–80. 10.1021/acs.energyfuels.9b00821.

[ref42] Hosseini-Moghadam S. M.-A.; Zahedi-Nejad A.; Bahrami M.; Torkaman M.; Ghayyem M.-A. Experimental and Modeling Investigations of Temperature Effect on Chemical Inhibitors of Asphaltene Aggregation. J. Pet Sci. Eng. 2021, 205, 108858 10.1016/j.petrol.2021.108858.

[ref43] Madhi M.; Kharrat R.; Hamoule T. Screening of Inhibitors for Remediation of Asphaltene Deposits: Experimental and Modeling Study. Petroleum 2018, 4, 168–77. 10.1016/j.petlm.2017.08.001.

[ref44] Kuang J.; Melendez-Alvarez A. A.; Yarbrough J.; Garcia-Bermudes M.; Tavakkoli M.; Abdallah D. S.; Punnapala S.; Vargas F. M. Assessment of the Performance of Asphaltene Inhibitors using a Multi-Section Packed Bed Column. Fuel 2019, 241, 247–54. 10.1016/j.fuel.2018.11.059.

[ref45] Wilson L.; Wilson M. J.; Green J.; Patey I. The Influence of Clay Mineralogy on Formation Damage in North Sea Reservoir Sandstones: A Review with Illustrative Examples. Earth Sci. Rev. 2014, 134, 70–80. 10.1016/j.earscirev.2014.03.005.

[ref46] Mohammed I.; Al Shehri D.; Mahmoud M.; Kamal M. S.; Alade O.; Arif M.; Patil S. Effect of Native Reservoir State and Oilfield Operations on Clay Mineral Surface Chemistry. Molecules (Basel, Switzerland) 2022, 27, 1739 10.3390/molecules27051739. 35268840 PMC8911921

[ref47] Frost R. L.; Kristof J. Raman and Infrared Spectroscopic Studies of Kaolinite Surfaces Modified by Intercalation. In Interface Science and Technology; Wypych F., Satyanarayana K. G., Eds.; Elsevier, 2004; Vol. 1, pp 184–215.

[ref48] Bougeard D.; Smirnov K. S.; Geidel E. Vibrational Spectra and Structure of Kaolinite: A Computer Simulation Study. J. Phys. Chem. B 2000, 104, 9210–7. 10.1021/jp0013255.

[ref49] Hu X. L.; Michaelides A. Water on the Hydroxylated (001) Surface of Kaolinite: From Monomer Adsorption to a Flat 2D Wetting Layer. Surf. Sci. 2008, 602, 960–74. 10.1016/j.susc.2007.12.032.

[ref50] Derakhshani-Molayousefi M.; McCullagh M. Deterring Effect of Resins on the Aggregation of Asphaltenes in n-Heptane. Energy Fuels 2020, 34, 16081–8. 10.1021/acs.energyfuels.0c03067.

[ref51] Kuznicki T.; Masliyah J. H.; Bhattacharjee S. Aggregation and Partitioning of Model Asphaltenes at Toluene-Water Interfaces: Molecular Dynamics Simulations. Energy Fuels 2009, 23, 5027–35. 10.1021/ef9004576.

[ref52] Kuznicki T.; Masliyah J. H.; Bhattacharjee S. Molecular Dynamics Study of Model Molecules Resembling Asphaltene-Like Structures in Aqueous Organic Solvent Systems. Energy Fuels 2008, 22, 2379–89. 10.1021/ef800057n.

[ref53] Morimoto M.; Fukatsu N.; Tanaka R.; Takanohashi T.; Kumagai H.; Morita T.; Tykwinski R. R.; Scott D. E.; Stryker J. M.; Gray M. R.; Sato T.; Yamamoto H. Determination of Hansen Solubility Parameters of Asphaltene Model Compounds. Energy Fuels 2018, 32, 11296–303. 10.1021/acs.energyfuels.8b02661.

[ref54] Mikami Y.; Liang Y.; Matsuoka T.; Boek E. S. Molecular Dynamics Simulations of Asphaltenes at the Oil-Water Interface: from Nanoaggregation to Thin-Film Formation. Energy Fuels 2013, 27, 1838–45. 10.1021/ef301610q.

[ref55] Silva F.; Guimarães M.; Seidl P.; Garcia M. Extraction and Characterization (compositional and thermal) of Asphaltenes from Brazilian Vacuum Residues. Braz J. Pet Gas 2013, 7, 107–18. 10.5419/bjpg2013-0009.

[ref56] Headen T. F.; Boek E. S.; Skipper N. T. Evidence for Asphaltene Nanoaggregation in Toluene and Heptane from Molecular Dynamics Simulations. Energy Fuels 2009, 23, 1220–9. 10.1021/ef800872g.

[ref57] Soulgani B. S.; Reisi F.; Norouzi F. Investigation into Mechanisms and Kinetics of Asphaltene Aggregation in Toluene/n-hexane Mixtures. Pet Sci. 2020, 17, 457–66. 10.1007/s12182-019-00383-3.

[ref58] Jorgensen W. L.; Maxwell D. S.; Tirado-Rives J. Development and Testing of the OPLS All-Atom Force Field on Conformational Energetics and Properties of Organic Liquids. J. Am. Chem. Soc. 1996, 118, 11225–36. 10.1021/ja9621760.

[ref59] Zhang L.; Greenfield M. L. Molecular Orientation in Model Asphalts Using Molecular Simulation. Energy Fuels 2007, 21, 1102–11. 10.1021/ef060449z.

[ref60] Headen T. F.; Hoepfner M. P. Predicting Asphaltene Aggregate Structure from Molecular Dynamics Simulation: Comparison to Neutron Total Scattering Data. Energy Fuels 2019, 33, 3787–95. 10.1021/acs.energyfuels.8b03196.

[ref61] Fu C.-F.; Tian S. X. A Comparative Study for Molecular Dynamics Simulations of Liquid Benzene. J. Chem. Theory Comput 2011, 7, 2240–52. 10.1021/ct2002122. 26606493

[ref62] Jorgensen W. L.; Laird E. R.; Nguyen T. B.; Tirado-Rives J. Monte Carlo Simulations of Pure Liquid Substituted Benzenes with OPLS Potential Functions. J. Comput. Chem. 1993, 14, 206–15. 10.1002/jcc.540140208.

[ref63] Cygan R. T.; Liang J.-J.; Kalinichev A. G. Molecular Models of Hydroxide, Oxyhydroxide, and clay phases and the development of a general force field. J. Phys. Chem. B 2004, 108, 1255–66. 10.1021/jp0363287.

[ref64] Bui T.; Phan A.; Cole D. R.; Striolo A. Transport Mechanism of Guest Methane in Water-Filled Nanopores. J. Phys. Chem. C 2017, 121, 15675–86. 10.1021/acs.jpcc.7b02713.

[ref65] Badmos S. B.; Bui T.; Striolo A.; Cole D. R. Factors Governing the Enhancement of Hydrocarbon Recovery via H2S and/or CO2 Injection: Insights from a Molecular Dynamics Study in Dry Nanopores. J. Phys. Chem. C 2019, 123, 23907–18. 10.1021/acs.jpcc.9b04247.

[ref66] Liu H.; Xiong H.; Yu H.; Wu K. Effect of Water Behaviour on the Oil Transport in Illite Nanopores: Insights from a Molecular Dynamics Study. J. Mol. Liq. 2022, 354, 118854 10.1016/j.molliq.2022.118854.

[ref67] Zhang L.; Lu X.; Liu X.; Yang K.; Zhou H. Surface Wettability of Basal Surfaces of Clay Minerals: Insights from Molecular Dynamics Simulation. Energy Fuels 2016, 30, 149–60. 10.1021/acs.energyfuels.5b02142.

[ref68] Li Y.; Zhang B.; Song H.; Yuan B.; Wang P.; Han S.; Zhu J.; Zhao Y. Effects of the Na+/Ca2+ Ratio and Cation Type in the Montmorillonite Interlayer on the Intercalated Methane Hydrate Formation: Insights from Molecular Dynamics Simulations. ACS Earth Space Chem. 2022, 6, 2745–54. 10.1021/acsearthspacechem.2c00264.

[ref69] Schampera B.; Solc R.; Woche S. K.; Mikutta R.; Dultz S.; Guggenberger G.; Tunega D. Surface Structure of Organoclays as Examined by X-ray Photoelectron Spectroscopy and Molecular Dynamics Simulations. Clay Miner 2015, 50, 353–67. 10.1180/claymin.2015.050.3.08. 27295321

[ref70] Ren K.; Liu S. The Effect of Surface Polarity on the Structure and Collective Dynamics of Liquid Ethanol. Phys. Chem. Chem. Phys. 2020, 22, 1204–13. 10.1039/C9CP05373E. 31848550

[ref71] Phan A.; Cole D. R.; Striolo A. Liquid Ethanol Simulated on Crystalline Alpha Alumina. J. Phys. Chem. B 2013, 117, 3829–40. 10.1021/jp312238d. 23484906

[ref72] Frenkel D.; Smit B. Understanding Molecular Simulation: From Algorithms to Applications; Elsevier, 2001.

[ref73] Darden T.; York D. M.; Pedersen L. G. Particle Mesh Ewald: An N·log(N) Method for Ewald Sums in Large Systems. J. Chem. Phys. 1993, 98, 10089 10.1063/1.464397.

[ref74] Hess B.; Kutzner C.; van der Spoel D.; Lindahl E. GROMACS 4: Algorithms for Highly Efficient, Load-Balanced, and Scalable Molecular Simulation. J. Chem. Theory Comput 2008, 4, 435–47. 10.1021/ct700301q. 26620784

[ref75] Van Der Spoel D.; Lindahl E.; Hess B.; Groenhof G.; Mark A. E.; Berendsen H. J. C. GROMACS: Fast, Flexible, and Free. J. Comput. Chem. 2005, 26, 1701–18. 10.1002/jcc.20291. 16211538

[ref76] Ren Z.; Cui J.; Qi K.; Yang G.; Chen Z.; Yang P.; Wang K. Control Effects of Temperature and Thermal Evolution History of Deep and Ultra-deep Layers on Hydrocarbon Phase State and Hydrocarbon Generation History. Nat. Gas Ind. B 2020, 7, 453–61. 10.1016/j.ngib.2020.09.003.

[ref77] Feyzullayev A.; Lerche I. Temperature-depth Control of Petroleum Occurrence in the Sedimentary Section of the South Caspian Basin. Pet Res. 2020, 5, 70–6. 10.1016/j.ptlrs.2019.10.003.

[ref78] Michael K.; Golab A.; Shulakova V.; Ennis-King J.; Allinson G.; Sharma S.; Aiken T. Geological Storage of CO2 in Saline Aquifers—A Review of the Experience from Existing Storage Operations. Int. J. Greenh Gas Control 2010, 4, 659–67. 10.1016/j.ijggc.2009.12.011.

[ref79] Liu J.; Sun L.; Li Z.; Wu X. Experimental Study on Reducing CO2-Oil Minimum Miscibility Pressure with Hydrocarbon Agents. Energies 2019, 12, 1975 10.3390/en12101975.

[ref80] Carauta A. N. M.; Seidl P. R.; Chrisman E. C. A. N.; Correia J. C. G.; Menechini P. d. O.; Silva D. M.; Leal K. Z.; de Menezes S. M. C.; de Souza W. F.; Teixeira M. A. G. Modeling Solvent Effects on Asphaltene Dimers. Energy Fuels 2005, 19, 1245–51. 10.1021/ef049809d.

[ref81] Schlitter J. Estimation of Absolute and Relative Entropies of Macromolecules using the Covariance Matrix. Chem. Phys. Lett. 1993, 215, 617–21. 10.1016/0009-2614(93)89366-P.

[ref82] Mallaki H.; Osfouri S.; Azin R. Analysis of Asphaltene Nano-aggregates Formation using Dynamic Light Scattering: Experimental and Kinetic Modeling. J. Dispers Sci. Technol. 2023, 44, 1147 10.1080/01932691.2021.2008418.

