# Supplementary material for: Understanding the Aggregation of Model Island and Archipelago Asphaltene Molecules near Kaolinite Surfaces using Molecular Dynamics
Source: Energy Fuels. 2023 Jul 28;37(16):11662–74. doi: 10.1021/acs.energyfuels.3c00504 (PMC10440792; doi:10.1021/acs.energyfuels.3c00504)
Supplement: Supplementary file 1 — ef3c00504_si_001.pdf [file ef3c00504_si_001.pdf]

Understanding the Aggregation of Model Island and Archipelago Asphaltene Molecules near Kaolinite Surfaces using Molecular Dynamics

Azeezat Ali[1], David R. Cole[2], Alberto Striolo[1, 3]*

[1]*Department of Chemical Engineering, University College London, London WC1E 6BT, United Kingdom* [2]*School of Earth Sciences, The Ohio State University, Columbus, Ohio 43210, United States of America* [3]*School of Chemical, Biological and Materials Engineering, The University of Oklahoma, Norman, Oklahoma 73019, United States of America*

Supplementary Information

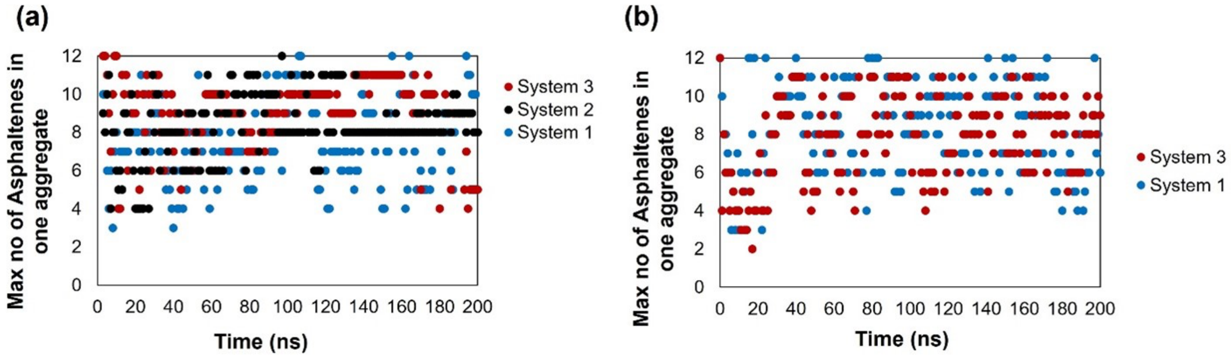

Figure S1. Maximum cluster size as a function of time for the island asphaltene (ASPH) on kaolinite surface at (a) 300 K and (b) 400 K. System compositions are shown in Table 1.

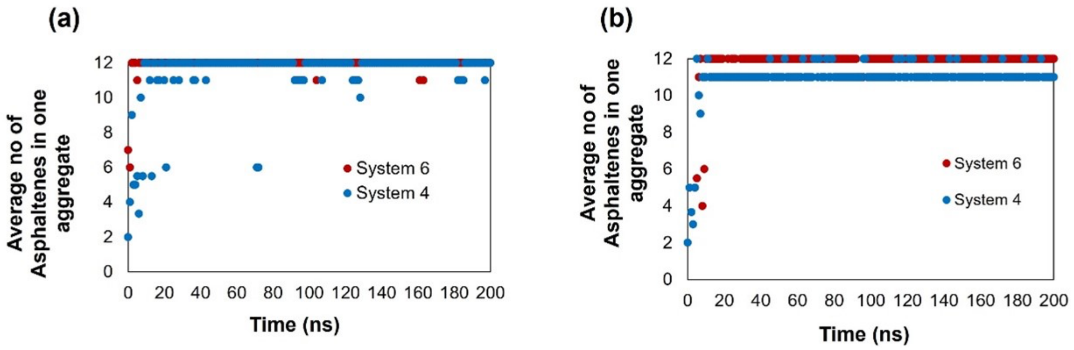

Figure S2. Average cluster size as a function of time for the archipelago asphaltene (ARCH) on (a) kaolinite surface at 400 K and (b) bulk systems at 300 K. System compositions are shown in Table 1. For clarity, the analysis for System 5 is not shown as the asphaltenes are also fully aggregated in this system.

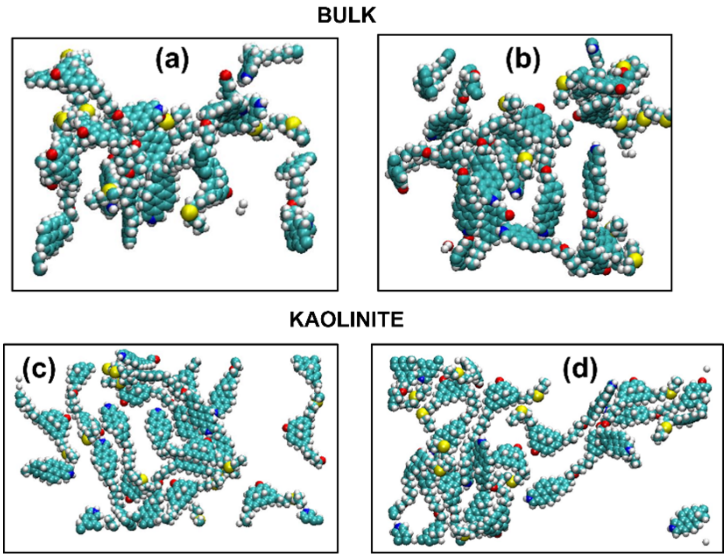

Figure S3. Simulation snapshots showing aggregation of ARCH asphaltenes for System 4 (panel a) and System 6 (panel b) in the bulk at 300 K and System 4 (panel c) and System 6 (panel d) on kaolinite surface at 400 K. System compositions are shown in Table 1.

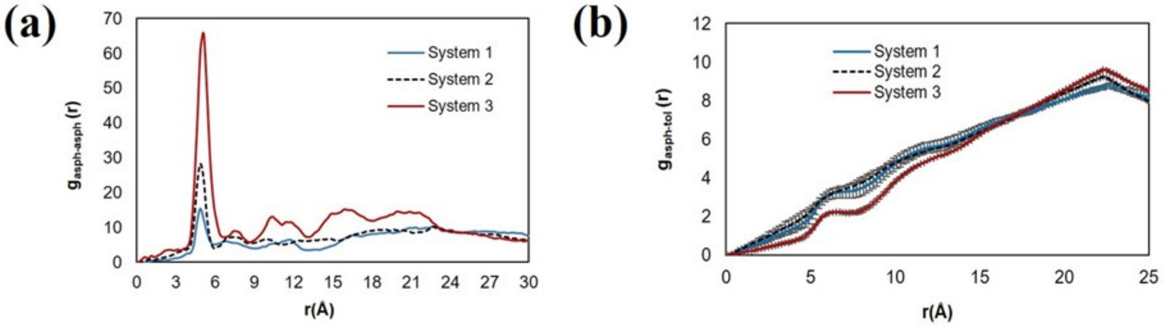

Figure S4. RDFs between (a) centre mass of the aromatic cores of ASPH asphaltene and (b) centre of mass of ASPH and toluene in bulk systems at 300 K. System compositions can be found in Table 1.

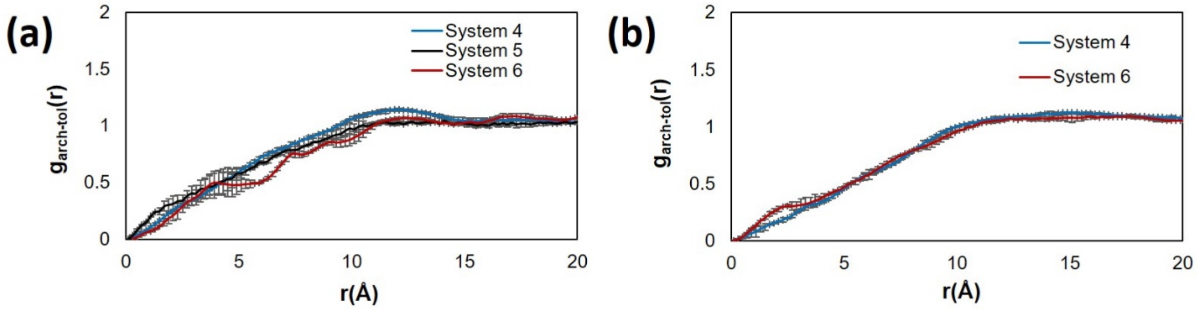

Figure S5. RDFs between the centre mass of archipelago asphaltene (ARCH) and the centre of mass of toluene on kaolinite surface at (a) 300 K and (b) 400 K. System compositions can be found in Table 1.

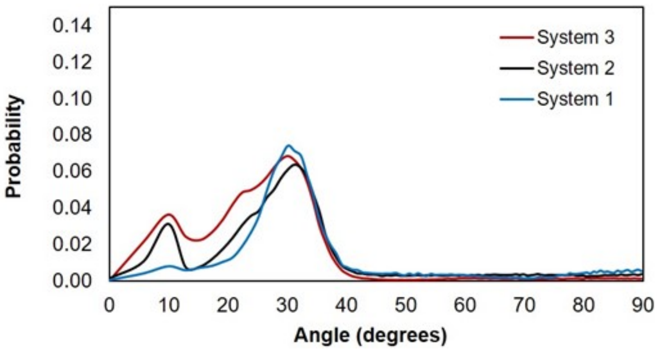

Figure S6. Angle between the poly-aromatic planes of island asphaltene pairs at 300 K in the bulk. System compositions can be found in Table 1.

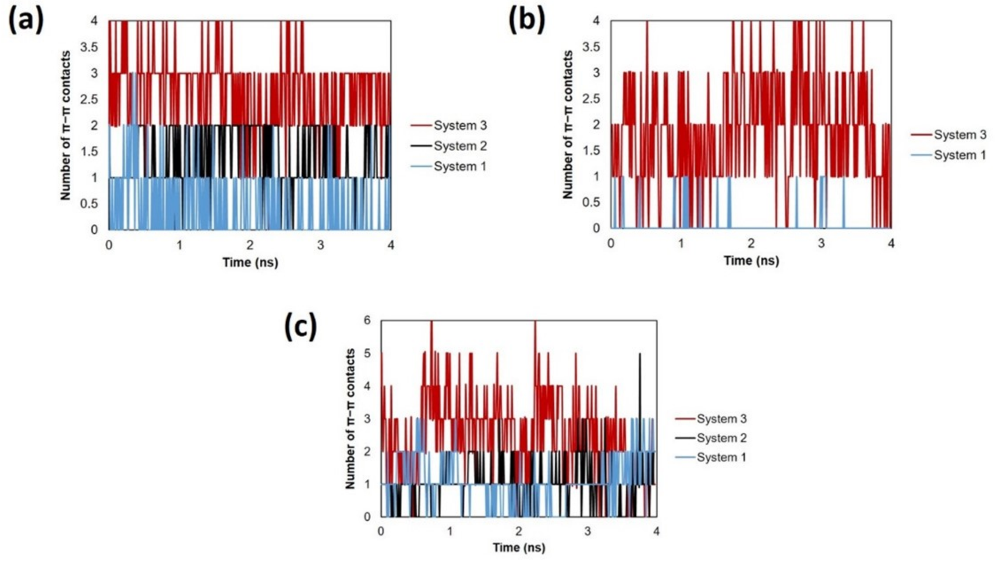

Figure S7. Number of π−π contacts between the aromatic cores of island asphaltene pairs in proximity of kaolinite at (a) 300 K (b) 400 K and in bulk systems at (c) 300 K.

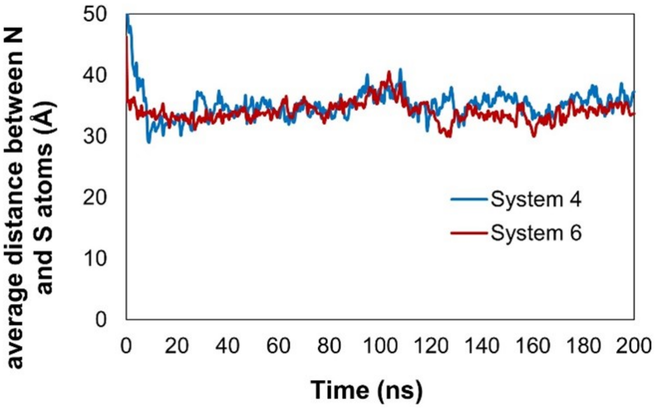

Figure S8. Distance between sulphur and nitrogen atoms for the archipelago asphaltene (ARCH) on kaolinite at 400 K. System compositions are shown in Table 1.

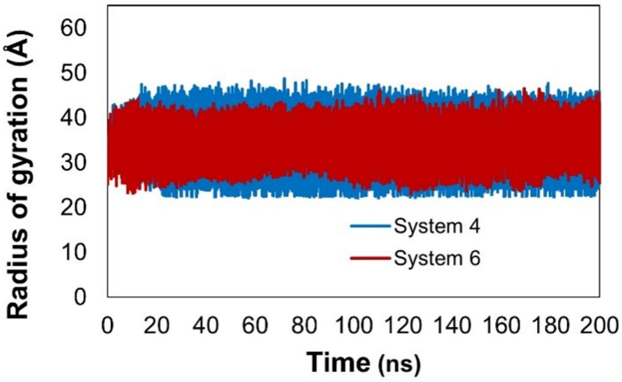

Figure S9. Radius of gyration of individual ARCH asphaltene in the bulk at 300 K. System compositions are shown in Table 1.

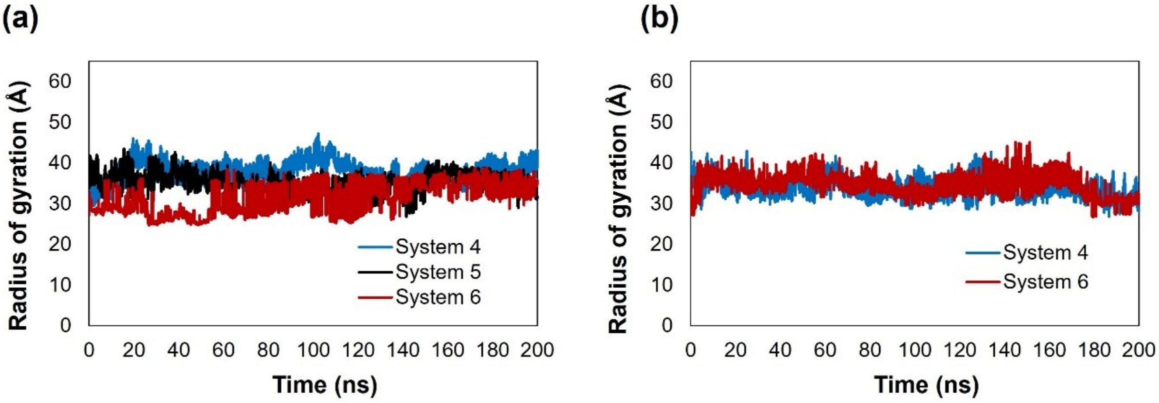

Figure S10. Radius of gyration of individual ARCH asphaltene on kaolinite at (a) 300 K and (b) 400 K. System compositions are shown in Table 1.

Table S1. Asphaltene-asphaltene interaction energies between archipelago asphaltenes on kaolinite surface and in bulk systems. System compositions can be found in Table 1.

	**Kaolinite at 300 K**		**Kaolinite at 400 K**		**Bulk at 300 K**	
**System**	**Asp-Asp (ΔE_{VDW})/ $kJmol^{-1}$**	**Asp-Asp (ΔE_{ELEC})/ $kJmol^{-1}$**	**Asp-Asp (ΔE_{VDW})/ $kJmol^{-1}$**	**Asp-Asp (ΔE_{ELEC})/ $kJmol^{-1}$**	**Asp-Asp (ΔE_{VDW})/ $kJmol^{-1}$**	**Asp-Asp (ΔE_{ELEC})/ $kJmol^{-1}$**
**4**	-1041 ± 49	1366 ± 1	-1282 ± 54	1265 ± 6	-1206 ± 129	1327 ± 46
**6**	-1193 ± 20	1345 ± 12	-1493 ± 71	1301 ± 5	-1337 ± 42	1251 ± 0.1
